# Volumetric Investigations on Molecular Interactions of Glycine/l-alanine in Aqueous Citric Acid Solutions at Different Temperatures

**DOI:** 10.1007/s10953-018-0829-6

**Published:** 2018-11-19

**Authors:** Poonam Patyar, Gurpreet Kaur, Tarnveer Kaur

**Affiliations:** Department of Chemistry, Punjabi University, Patiala, Punjab 147 002 India

**Keywords:** Hydration number, Interaction coefficients, Partial molar expansibilities, Partial molar volumes, Partial molar volumes of transfer, Apparent specific volumes

## Abstract

Apparent molar volumes $(\phi_{V})$ of glycine/l-alanine in water and in aqueous citric acid (CA) solutions of varying concentrations, *i.e.* (0.05, 0.10, 0.20, 0.30, 0.40 and 0.50) mol·kg^−1^ were determined from density measurements at temperatures *T* = (288.15, 298.15, 308.15, 310.15 and 318.15) K and at atmospheric pressure. Limiting partial molar volumes $(\phi_V^{\text{o}})$ and their corresponding partial molar volumes of transfer $(\Delta_{\text{tr}} \phi_{V} )$ have been calculated from the $\phi_{V}$ data. The negative $\Delta_{\text{tr}} \phi_{V}$ values obtained for glycine/l-alanine from water to aqueous CA solutions indicate the dominance of hydrophilic–hydrophobic/hydrophobic–hydrophilic and hydrophobic–hydrophobic interactions over ion/hydrophilic–dipolar interactions. Further, pair and triplet interaction coefficients, *i.e.* $(V_{\text{AB}} )\;{\text{and}}\; (V_{\text{ABB}} )$ along with hydration number $(n_{\text{H}} )$ have also been calculated. The effect of temperature on the volumetric properties of glycine/l-alanine in water and in aqueous CA solutions has been determined from the limiting partial molar expansibilities $(\partial \phi_{V}^{\text{o}} /\partial T)_{p}$ and their second-order derivative $(\partial^{2} \phi_{V}^{\text{o}} /\partial T^{2} )_{{P}}$. The apparent specific volumes $(\nu_{\phi} )$ for glycine and l-alanine tend to approach sweet taste behavior both in the presence of water and in aqueous CA solutions. The $\nu_{\phi}$ values for glycine/l-alanine increase with increase in concentration of CA at all temperatures studied. This reveals that CA helps in enhancing the sweet taste behavior of glycine/l-alanine which also supports the dominance of hydrophobic–hydrophobic interactions.

**Electronic supplementary material:**

The online version of this article (10.1007/s10953-018-0829-6) contains supplementary material, which is available to authorized users.

## Introduction

Amino acids are the building blocks of proteins, thus they are regarded as an ideal model for the study of protein functioning and their complex structure [1, 2]. The functional properties of protein molecules depend upon their three dimensional structure which arises due to a particular sequence of amino acids (hereafter written as AA) in a polypeptide chain. All AAs exists as zwitterionic species in aqueous solutions [3], thus their thermodynamic properties in a variety of media can provide valuable information about the stability and denaturation of proteins [4–16]. Recently, Lomesh and Kumar [17, 18] have reported the volumetric and acoustic properties of glycine, diglycine, l-alanine and l-phenylalanine in water and in 0.1 mol·kg^−1^ aqueous citric acid at different temperatures. Further, a literature survey reveals that not much systematic data is available regarding the thermodynamic and transport properties of AA as a function of concentration in citric acid solutions at different temperatures.

Citric acid (CA) (2-hydroxy-1,2,3-propanetricarboxylic acid) is a tri-basic acid and common metabolite of plants and animals. It is an environmentally acceptable organic acid, used in food, beverages, pH adjustment in buffers, and pharmaceuticals (as an acidifier) [19, 20], and is known to increase the stability of proteins [13]. The presence of one hydroxyl and three carboxyl groups in CA provides effective chemical properties so that it can act as an important metabolite in the citric acid cycle (CAC) of all aerobic organisms [21]. It also acts as a precursor for the bio-synthesis of many compounds in CAC including AA [22]. Therefore, it is of great interest to investigate the molecular interactions of AA with CA which can influence the behavior and conformational stability of proteins. In light of the above facts, presently we report the apparent molar volumes $(\phi_{V} )$ of glycine/l-alanine (both are non-essential amino acids) in water and in aqueous CA solutions, $m_{\text{c}}$ (molality of aqueous CA) ≈ (0.05, 0.10, 0.20, 0.30, 0.40 and 0.50) mol·kg^−1^ at temperatures, *T *= (288.15, 298.15, 308.15, 310.15 and 318.15) K and at atmospheric pressure, obtained from experimental densities. Partial molar volumes $(\phi_{V}^{\text{o}} )$ calculated from $\phi_{V}$ data have been used to calculate partial molar volumes of transfer $(\Delta_{\text{tr}} \phi_{V} ),$ apparent specific volumes $(\nu_{\phi} )$, pair $(V_{\text{AB}} )$ and triplet $(V_{\text{ABB}} )$ interaction coefficients, partial molar expansibilities $(\partial \phi_{V}^{\text{o}} /\partial T)_{p}$, their second order derivatives $(\partial^{2} \phi_{V}^{\text{o}} /\partial T^{2} )_{p}$ and hydration number $(n_{\text{H}} )$. The volumetric behavior of glycine in aqueous CA solutions (present work) are compared with glycine in aqueous succinic acid (SA) solutions, reported earlier from our laboratory [23].

## Experimental Section

### Chemicals Used

Glycine (C_2_H_5_NO_2_), l-alanine (C_3_H_7_NO_2_) and citric acid (C_6_H_8_O_7_) of analytical grade with mass fraction purity ≥ 99% were procured from S. D. Fine Chemical Ltd. (SDFCL), India. Specifications of the chemicals used are given in Table 1. All the chemicals were used without any further purification; however, they were dried in a vacuum oven for 24 h at *T* = 318.15 K, and then kept in a vacuum desiccator over anhydrous CaCl_2_ prior to their use.

**Table 1:** Specifications of the chemicals used in present work

Chemical name	Molecular formula	Structure	Molecular weight ($10^{ - 3}$, kg·${\text{mol}}^{ - 1}$)	CAS no.	Source	Mass fraction purity (%)
Glycine	${\text{C}}_{2} {\text{H}}_{5} {\text{NO}}_{2}$		75.07	56-40-6	SDFCL	≥ 99
l-Alanine	${\text{C}}_{3} {\text{H}}_{7} {\text{NO}}_{2}$		89.09	56-41-7	SDFCL	≥ 99
Ctric acid, anhydrous	${\text{C}}_{6} {\text{H}}_{8} {\text{O}}_{7}$		192.13	77-92-9	SDFCL	≥ 99

**Fig. 1:**
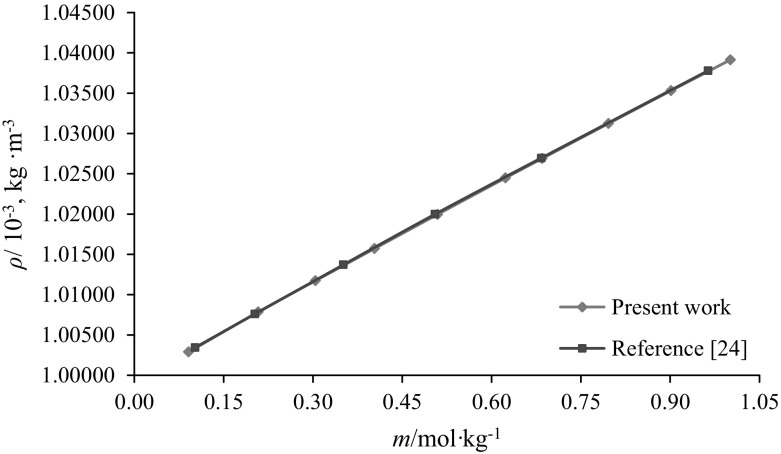
Representative plots of densities $\left( \rho \right)$ versus molality $\left( m \right)$: (blue filled square, present work; red filled square, literature values [24] of NaCl solutions at 288.15 K (Color figure online)

Deionized, double distilled and degassed water with specific conductance < 1 × 10^−4^ S·m^−1^ was used to prepare all the solutions. The pHs of the experimental solutions were checked using a pH meter (Systronics digital pH meter-335, India). The standard deviation obtained for the whole set of experimental data is ± 0.02 pH unit. Accuracy in pH measurements was checked by calibrating the pH meter using standard buffer solutions of pH 7.00 and pH 9.20. The pHs of the stock solutions, *i.e.* at all concentrations of aqueous citric acid solutions, lie between 2.08 and 2.44, in the case of glycine in aqueous citric acid solutions the pHs lie from 1.96 to 3.61 and for l-alanine in aqueous citric acid solutions it varies from 1.99 to 3.94. Solutions were prepared on the molality basis using a Citizen CY-204 balance having a precision of ± 0.1 mg. The overall uncertainty in molality was estimated to be < 5 × 10^−3^ mol·kg^−1^. Solution densities were measured using a vibrating-tube digital density meter (DMA 4500 M from Anton Paar, Austria). The sensitivity of the instrument corresponds to a precision in density measurements of ± 1 × 10^−2^ kg·m^−3^ and accuracy of ± 5 × 10^−2^ kg·m^−3^, respectively. The density meter has a built in thermostat to maintain the desired temperatures within ± 0.01 K and was calibrated with double distilled and degassed water before each series of experiments. The performance of the density meter was checked by measuring the densities of aqueous sodium chloride (NaCl) solutions, which agree well with the literature values [24] as shown in Fig. 1.

## Results and Discussion

### Volumetric Properties

**Fig. 2:**
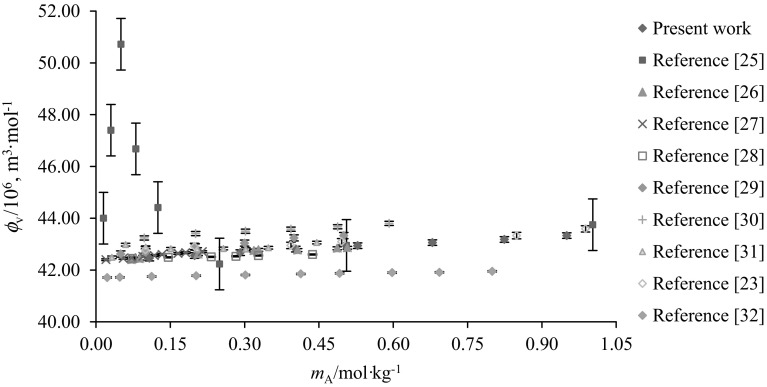
Representative plots of apparent molar volumes $\left( {\phi_{V} } \right)$ versus molality $\left( {m_{\text{A}} } \right)$ of glycine in water at 288.15 K: (red filled square, present work and blue filled square, literature values [23, 25–32]) (Color figure online)

**Fig. 3:**
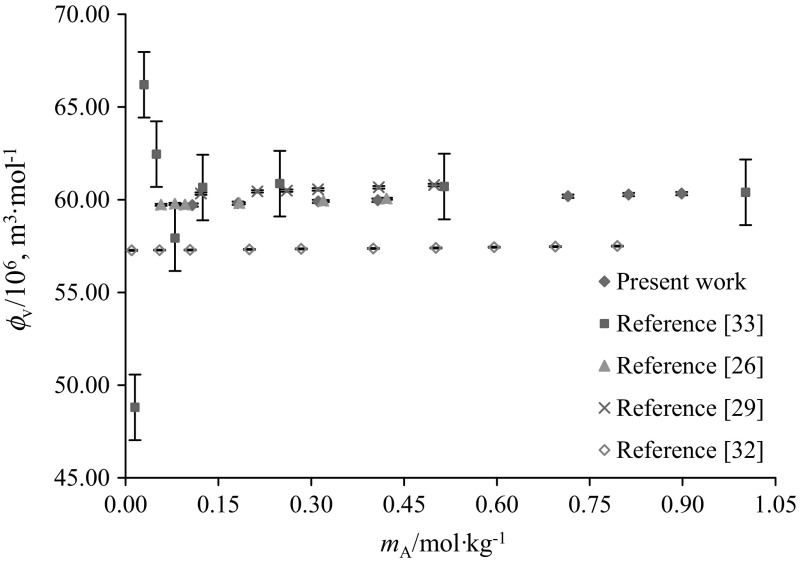
Representative plots of apparent molar volumes $\left( {\phi_{V} } \right)$ versus molality $\left( {m_{\text{A}} } \right)$ of l-alanine in water at 288.15 K: red filled square, present work and blue filled square, literature values [26, 29, 32, 33] (Color figure online)

**Fig. 4:**
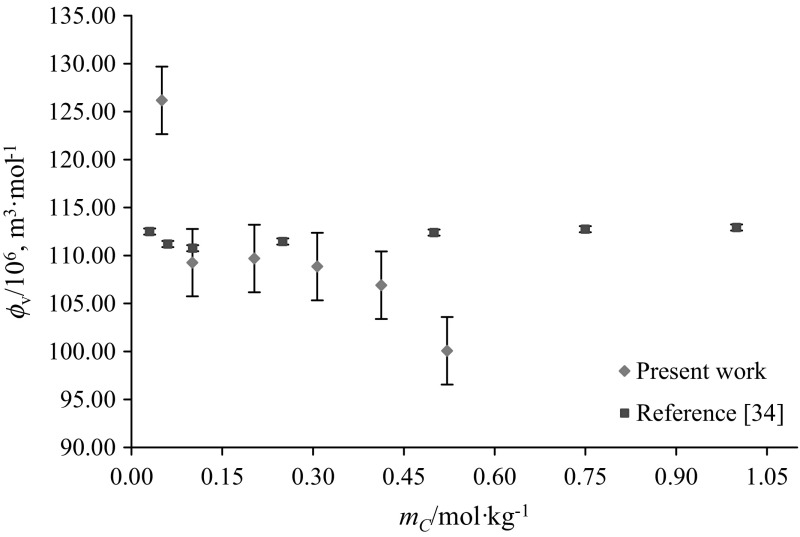
Representative plots of apparent molar volumes $\left( {\phi_{V} } \right)$ versus molality $\left( {m_{\text{c}} } \right)$ of citric acid in water at 288.15 K: red filled square, Present work and blue filled square literature values [34] (Color figure online)

The apparent molar volumes $(\phi_{\text{V}} )$ of glycine and l-alanine in water and in varying concentrations of aqueous CA solutions were calculated from experimental solution densities $(\rho )$ at temperatures *T* = (288.15, 298.15, 308.15, 310.15 and 318.15) K and at atmospheric pressure, by employing the following equation:

1 $$\phi_{V} = M/\rho - [1000(\rho - \rho_{\text{o}} )]/m_{\text{A}} \rho \rho_{\text{o}}$$

where *M* (kg·mol^−1^) is the molar mass of the glycine/l-alanine, *m*_A_ (mol·kg^−1^) is the molality of glycine/l-alanine, $\rho_{\text{o}}$ and $\rho$ are the densities of the solvent (water or water + CA) and solution (water + CA + glycine/l-alanine), respectively. The $\phi_{V}$ values of glycine/l-alanine along with $\rho_{\text{o}}$ and $\rho$ as a function of molality, in water and in aqueous CA solutions at different temperatures, are summarized in Table 2. The standard uncertainty in the apparent molar volume due to molality u(*m*) and density u(*ρ*) has been calculated and is (≤ 0.0960 and ≤ 1.611 × 10^−6^ m^3^·mol^−1^), respectively. Representative plots of $\phi_{V}$ values for the presently studied systems (*i.e.* glycine, l-alanine and citric acid in water) at *T* = 288.15 K agree well with earlier reported data [25–34] and are shown in Figs. 2, 3 and 4. The values reported by the authors [25, 33] are higher (at lower concentrations) than to our present results as well as with the other literature data. (The standard uncertainty in molality, u(*m*) and apparent molar volume, u($\phi_{V}$) of (succinic acid + glycine) system are ≤ 2.21 × 10^−4^ mol·kg^−1^ and (0.03–0.65) × 10^−6^ m^3^·mol^−1^, respectively).

**Table 2:** Densities $\left( \rho \right)$ and apparent molar volumes $\left( {\phi_{{v}} } \right)$ of glycine and l-alanine in water and in aqueous CA solutions at *T* = (288.15, 298.15, 308.15, 310.15 and 318.15) K and *p* = 101.3 kPa

$m_{\text{A}}$ $\left( {{\text{mol}} {\cdot} {\text{kg}}^{ - 1} } \right)$	$T(\text{K}) \rightarrow 288.15$		298.15		308.15		310.15		318.15	
	$$\rho$$ $$\times$$ 10^−3^ $$\left( {{\text{kg}} {\cdot} {\text{m}}^{ - 3} } \right)$$	$$\phi_{V}$$$$\times$$ 10^6^ $$\left( {{\text{m}}^{3} {\cdot} {\text{mol}}^{ - 1} } \right)$$	$$\rho$$ $$\times$$ 10^−3^ $$\left( {{\text{kg}} {\cdot} {\text{m}}^{ - 3} } \right)$$	$$\phi_{V}$$ $$\times$$ 10^6^ $$\left( {{\text{m}}^{3} {\cdot }{\text{mol}}^{ - 1} } \right)$$	$$\rho$$ $$\times$$ 10^−3^ $$\left( {{\text{kg}} {\cdot} {\text{m}}^{ - 3} } \right)$$	$$\phi_{V}$$ $$\times$$ 10^6^ $$\left( {{\text{m}}^{3} {\cdot} {\text{mol}}^{ - 1} } \right)$$	$$\rho$$ $$\times$$ 10^−3^ $$\left( {{\text{kg}} {\cdot} {\text{m}}^{ - 3} } \right)$$	$$\phi_{V}$$ $$\times$$ 10^6^ $$\left( {{\text{m}}^{3} {\cdot} {\text{mol}}^{ - 1} } \right)$$	$$\rho$$ $$\times$$ 10^−3^ $$\left( {{\text{kg}} {\cdot }{\text{m}}^{ - 3} } \right)$$	$$\phi_{V}$$ $$\times$$ 10^6^ $$\left( {{\text{m}}^{3} {\cdot} {\text{mol}}^{ - 1} } \right)$$
Glycine in water										
0.0000	0.99909		0.99704		0.99403		0.99332		0.99021	
0.1078	1.00259	42.46	1.00046	43.22	0.99738	43.91	0.99667	43.92	0.99353	44.25
0.1991	1.00551	42.56	1.00331	43.34	1.00018	43.99	0.99947	44.00	0.99631	44.29
0.2911	1.00841	42.67	1.00614	43.45	1.00296	44.08	1.00224	44.13	0.99907	44.38
0.4034	1.01189	42.81	1.00954	43.58	1.00632	44.14	1.00559	44.20	1.00239	44.47
0.5282	1.01569	42.94	1.01326	43.69	1.01000	44.21	1.00926	44.28	1.00602	44.57
0.6791	1.02022	43.06	1.01769	43.80	1.01437	44.30	1.01362	44.37	1.01035	44.66
0.8244	1.02448	43.18	1.02186	43.91	1.01849	44.39	1.01773	44.46	1.01443	44.75
0.9504	1.02807	43.33	1.02539	44.04	1.02199	44.50	1.02119	44.60	1.01756	44.89
Glycine in 0.05 $\left( {{\text{mol}}{\cdot}{\text{kg}}^{ - 1} } \right)$ CA										
0.0000	1.00237		1.00023		0.99715		0.99643		0.99328	
0.1981	1.00876	42.52	1.00647	43.30	1.00330	43.79	1.00255	43.95	0.99935	44.25
0.3022	1.01205	42.62	1.00967	43.42	1.00646	43.89	1.00570	44.04	1.00247	44.35
0.4196	1.01570	42.70	1.01322	43.54	1.00997	43.99	1.00919	44.14	1.00593	44.45
0.5062	1.01835	42.79	1.01581	43.61	1.01248	44.15	1.01172	44.23	1.00844	44.54
0.6103	1.02149	42.89	1.01886	43.73	1.01550	44.23	1.01473	44.33	1.01142	44.64
0.7179	1.02470	42.98	1.02200	43.79	1.01859	44.30	1.01783	44.36	1.01448	44.69
0.7599	1.02591	43.05	1.02319	43.85	1.01976	44.36	1.01900	44.42	1.01566	44.72
0.8538	1.02956	43.16	1.02677	43.93	1.02330	44.42	1.02251	44.51	1.01912	44.82
Glycine in 0.10 $\left( {{\text{mol}}{\cdot}{\text{kg}}^{ - 1} } \right)$ CA										
0.0000	1.00729		1.00503		1.00186		1.00111		0.99790	
0.2258	1.01458	42.39	1.01217	43.24	1.00886	43.73	1.00810	43.79	1.00484	44.05
0.3080	1.01719	42.43	1.01465	43.36	1.01136	43.82	1.01058	43.89	1.00728	44.23
0.4154	1.02053	42.56	1.01791	43.44	1.01454	43.94	1.01378	44.00	1.01046	44.31
0.5221	1.02380	42.66	1.02110	43.53	1.01769	44.03	1.01689	44.13	1.01357	44.39
0.6159	1.02665	42.73	1.02383	43.66	1.02038	44.16	1.01960	44.22	1.01624	44.51
0.7295	1.03005	42.81	1.02715	43.71	1.02362	44.25	1.02283	44.32	1.01943	44.63
0.8388	1.03324	42.93	1.03027	43.81	1.02669	44.34	1.02591	44.39	1.02246	44.73
0.9371	1.03609	43.01	1.03303	43.89	1.02994	44.39	1.02860	44.49	1.02515	44.80
Glycine in 0.20 $\left( {{\text{mol}}{\cdot}{\text{kg}}^{ - 1} } \right)$ CA										
0.0000	1.01523		1.01277		1.00943		1.00866		1.00533	
0.0859	1.01803	42.19	1.01552	42.78	1.01214	43.28	1.01137	43.29	1.00803	43.44
0.1891	1.02136	42.24	1.01878	42.89	1.01535	43.40	1.01457	43.46	1.01121	43.60
0.3102	1.02520	42.34	1.02251	43.10	1.01903	43.58	1.01825	43.62	1.01487	43.83
0.4066	1.02819	42.48	1.02540	43.30	1.02191	43.70	1.02112	43.81	1.01769	44.05
0.5207	1.03169	42.58	1.02880	43.42	1.02522	43.92	1.02441	44.01	1.02097	44.26
0.5506	1.03256	42.68	1.02968	43.46	1.02608	43.97	1.02527	44.05	1.02182	44.31
0.6985	1.03700	42.78	1.03393	43.67	1.03026	44.18	1.02945	44.25	1.02598	44.50
0.8041	1.04011	42.87	1.03695	43.76	1.03322	44.29	1.03240	44.36	1.02892	44.60
0.9182	1.04342	42.96	1.04016	43.85	1.03639	44.37	1.03559	44.44	1.03202	44.72
Glycine in 0.30 $\left( {{\text{mol}}{\cdot}{\text{kg}}^{ - 1} } \right)$ CA										
0.0000	1.02329		1.02064		1.01710		1.01631		1.01289	
0.1008	1.02658	42.06	1.02388	42.57	1.02030	42.99	1.01949	43.19	1.01605	43.43
0.1934	1.02956	42.15	1.02676	42.72	1.02317	43.22	1.02235	43.38	1.01890	43.57
0.2606	1.03169	42.23	1.02882	42.89	1.02521	43.38	1.02438	43.54	1.02092	43.73
0.3647	1.03495	42.34	1.03196	43.06	1.02835	43.51	1.02749	43.70	1.02401	43.91
0.5281	1.03999	42.47	1.03680	43.21	1.03316	43.72	1.03232	43.82	1.02871	44.22
0.5944	1.04199	42.54	1.03866	43.32	1.03503	43.88	1.03419	43.97	1.03061	44.28
0.6569	1.04386	42.60	1.04042	43.46	1.03677	44.01	1.03592	44.11	1.03234	44.40
0.7337	1.04614	42.67	1.04260	43.58	1.03887	44.18	1.03805	44.23	1.03445	44.52
0.8901	1.05073	42.77	1.04723	43.74	1.04322	44.30	1.04238	44.37	1.03873	44.68
Glycine in 0.40 $\left( {{\text{mol}}{\cdot}{\text{kg}}^{ - 1} } \right)$ CA										
0.0000	1.03179		1.02894		1.02525		1.02443		1.02089	
0.3118	1.04192	41.83	1.03898	42.14	1.03519	42.48	1.03424	42.89	1.03062	43.18
0.4341	1.04574	42.01	1.04279	42.26	1.03898	42.56	1.03798	42.97	1.03432	43.28
0.5122	1.04814	42.11	1.04518	42.34	1.04138	42.59	1.04032	43.05	1.03664	43.36
0.5820	1.05023	42.24	1.04727	42.45	1.04350	42.63	1.04239	43.12	1.03869	43.43
0.6992	1.05373	42.38	1.05079	42.54	1.04703	42.68	1.04584	43.20	1.04212	43.50
0.7254	1.05447	42.45	1.05155	42.58	1.04779	42.72	1.04659	43.24	1.04285	43.55
0.9271	1.06035	42.64	1.05741	42.77	1.05372	42.82	1.05541	43.34	1.04865	43.62
Glycine in 0.50 $\left( {{\text{mol}}{\cdot}{\text{kg}}^{ - 1} } \right)$ CA										
0.0000	1.04293		1.03988		1.03601		1.03516		1.03150	
0.0981	1.04617	41.50	1.04309	41.81	1.03918	42.33	1.03830	42.53	1.03459	43.05
0.2034	1.04959	41.61	1.04647	41.96	1.04251	42.42	1.04160	42.61	1.03784	43.22
0.2978	1.05261	41.71	1.04945	42.12	1.04547	42.48	1.04456	42.68	1.04046	43.39
0.4130	1.05622	41.86	1.05297	42.35	1.04909	42.53	1.04810	42.75	1.04411	43.55
0.4988	1.05887	41.96	1.05554	42.52	1.05164	42.62	1.05070	42.80	1.04662	43.65
0.6104	1.06225	42.10	1.05885	42.67	1.05499	42.71	1.05402	42.90	1.04982	43.79
0.6678	1.06397	42.16	1.06052	42.76	1.05669	42.76	1.05571	42.95	1.05144	43.87
l-Alanine in water										
0.0000	0.99909		0.99704		0.99403		0.99332		0.99021	
0.1078	1.00224	59.71	1.00012	60.43	0.99707	60.90	0.99635	61.02	0.99323	61.22
0.1818	1.00436	59.81	1.00220	60.48	0.99912	60.97	0.99840	61.05	0.99527	61.27
0.3114	1.00802	59.91	1.00580	60.52	1.00267	61.02	1.00194	61.10	0.99877	61.41
0.4077	1.01069	59.97	1.00841	60.61	1.00525	61.08	1.00452	61.16	1.00132	61.49
0.7149	1.01892	60.19	1.01649	60.80	1.01323	61.26	1.01247	61.36	1.00924	61.64
0.8130	1.02144	60.28	1.01896	60.89	1.01567	61.35	1.01491	61.44	1.01166	61.73
0.8986	1.02363	60.33	1.02112	60.92	1.01779	61.40	1.01704	61.47	1.01369	61.85
l-Alanine in 0.05 $\left( {{\text{mol}}{\cdot}{\text{kg}}^{ - 1} } \right)$ CA										
0.0000	1.00484		1.00268		0.99958		0.99885		0.99569	
0.1330	1.00871	59.61	1.00654	59.76	1.00344	59.85	1.00270	59.95	0.99953	60.12
0.1973	1.01054	59.71	1.00837	59.83	1.00527	59.93	1.00453	60.00	1.00135	60.20
0.2631	1.01239	59.80	1.01021	59.94	1.00711	60.03	1.00637	60.10	1.00318	60.31
0.4041	1.01628	59.94	1.01407	60.13	1.01098	60.21	1.01023	60.28	1.00705	60.43
0.4940	1.01871	60.02	1.01648	60.23	1.01339	60.31	1.01263	60.40	1.00945	60.54
0.5800	1.02100	60.10	1.01874	60.34	1.01566	60.41	1.01489	60.50	1.01173	60.60
0.6971	1.02406	60.20	1.02177	60.46	1.01867	60.56	1.01791	60.63	1.01476	60.72
0.7833	1.02626	60.29	1.02396	60.54	1.02088	60.62	1.02011	60.70	1.01693	60.83
0.9042	1.02933	60.37	1.02696	60.67	1.02387	60.76	1.02255	60.82	1.01994	60.94
l-Alanine in 0.10 $\left( {{\text{mol}}{\cdot}{\text{kg}}^{ - 1} } \right)$ CA										
0.0000	1.00978		1.00752		1.00433		1.00358		1.00036	
0.1073	1.01290	59.53	1.01064	59.60	1.00745	59.70	1.00669	59.81	1.00347	59.91
0.1924	1.01533	59.61	1.01307	59.68	1.00987	59.83	1.00911	59.91	1.00588	60.06
0.2994	1.01833	59.72	1.01606	59.83	1.01288	59.89	1.01210	60.02	1.00886	60.19
0.3970	1.02101	59.82	1.01875	59.89	1.01555	60.02	1.01478	60.09	1.01151	60.32
0.4955	1.02366	59.93	1.02139	60.02	1.01820	60.13	1.01742	60.21	1.01414	60.44
0.6009	1.02645	60.03	1.02418	60.12	1.02096	60.27	1.02020	60.31	1.01692	60.52
0.7062	1.02920	60.10	1.02692	60.21	1.02371	60.34	1.02293	60.40	1.01965	60.60
0.8129	1.03190	60.22	1.02963	60.31	1.02643	60.42	1.02562	60.52	1.02234	60.70
0.9539	1.03542	60.33	1.03315	60.42	1.02993	60.56	1.02915	60.61	1.02577	60.89
l-Alanine in 0.20 $\left( {{\text{mol}}{\cdot}{\text{kg}}^{ - 1} } \right)$ CA										
0.0000	1.01781		1.01535		1.01197		1.01120		1.00789	
0.0990	1.02068	59.39	1.01821	59.56	1.01483	59.67	1.01406	59.69	1.01075	59.80
0.1844	1.02310	59.53	1.02063	59.66	1.01725	59.76	1.01648	59.79	1.01316	59.94
0.3128	1.02669	59.61	1.02420	59.78	1.02083	59.85	1.02005	59.91	1.01670	60.14
0.3873	1.02872	59.70	1.02623	59.85	1.02286	59.93	1.02207	60.01	1.01871	60.24
0.4935	1.03157	59.81	1.02909	59.93	1.02571	60.04	1.02491	60.12	1.02155	60.33
0.5962	1.03428	59.90	1.03177	60.06	1.02841	60.13	1.02758	60.26	1.02424	60.42
0.7044	1.03708	59.99	1.03458	60.12	1.03121	60.22	1.03037	60.34	1.02699	60.55
0.7844	1.03906	60.12	1.03660	60.21	1.03321	60.33	1.03239	60.42	1.02900	60.63
0.8932	1.04177	60.22	1.03929	60.32	1.03591	60.43	1.03506	60.55	1.03169	60.73
l-Alanine in 0.30 $\left( {{\text{mol}}{\cdot}{\text{kg}}^{ - 1} } \right)$ CA										
0.0000	1.02690		1.02424		1.02072		1.01991		1.01647	
0.0858	1.02937	59.30	1.02671	59.38	1.02319	59.49	1.02237	59.63	1.01893	59.74
0.1864	1.03222	59.39	1.02955	59.52	1.02603	59.63	1.02521	59.71	1.02177	59.82
0.2857	1.03498	59.47	1.03230	59.62	1.02879	59.70	1.02795	59.82	1.02452	59.90
0.4147	1.03849	59.58	1.03580	59.73	1.03230	59.80	1.03146	59.90	1.02802	60.01
0.5290	1.04153	59.68	1.03883	59.84	1.03534	59.90	1.03449	60.00	1.03104	60.13
0.6150	1.04377	59.76	1.04106	59.93	1.03758	59.98	1.03671	60.10	1.03325	60.25
0.7023	1.04600	59.85	1.04328	60.02	1.03980	60.08	1.03892	60.21	1.03546	60.35
0.7884	1.04817	59.93	1.04544	60.11	1.04195	60.18	1.04107	60.30	1.03759	60.46
0.8430	1.04952	59.99	1.04676	60.19	1.04329	60.25	1.04239	60.38	1.03892	60.53
l-Alanine in 0.40 $\left( {{\text{mol}}{\cdot}{\text{kg}}^{ - 1} } \right)$ CA										
0.0000	1.03479		1.03193		1.02825		1.02741		1.02385	
0.1711	1.03965	59.29	1.03679	59.44	1.03311	59.55	1.03226	59.64	1.02870	59.75
0.2959	1.04310	59.39	1.04022	59.55	1.03655	59.66	1.03569	59.76	1.03213	59.84
0.3711	1.04513	59.48	1.04225	59.63	1.03857	59.77	1.03771	59.85	1.03416	59.91
0.4846	1.04815	59.58	1.04526	59.74	1.04157	59.87	1.04071	59.94	1.03716	60.03
0.5712	1.05039	59.69	1.04751	59.82	1.04382	59.95	1.04294	60.05	1.03937	60.18
0.7049	1.05381	59.80	1.05093	59.92	1.04724	60.05	1.04635	60.15	1.04276	60.31
0.9162	1.05905	59.93	1.05615	60.10	1.05246	60.23	1.05157	60.31	1.04797	60.48
l-Alanine in 0.50 $\left( {{\text{mol}}{\cdot}{\text{kg}}^{ - 1} } \right)$ CA										
0.0000	1.04164		1.03860		1.03475		1.03389		1.03024	
0.0825	1.04399	59.14	1.04095	59.23	1.03710	59.36	1.03624	59.38	1.03259	59.50
0.1808	1.04674	59.24	1.04370	59.34	1.03985	59.46	1.03898	59.54	1.03533	59.66
0.2848	1.04959	59.34	1.04656	59.41	1.04270	59.56	1.04183	59.62	1.03818	59.74
0.3801	1.05215	59.44	1.04911	59.54	1.04527	59.64	1.04439	59.72	1.04074	59.84
0.4973	1.05525	59.53	1.05221	59.63	1.04837	59.73	1.04748	59.82	1.04384	59.92
0.6030	1.05797	59.64	1.05493	59.74	1.05110	59.83	1.05021	59.91	1.04657	60.01
0.7018	1.06045	59.75	1.05738	59.89	1.05359	59.93	1.05269	60.02	1.04905	60.13
0.7889	1.06259	59.85	1.05952	59.99	1.05574	60.03	1.05484	60.11	1.05117	60.25
0.8216	1.06337	59.90	1.06031	60.03	1.05652	60.09	1.05559	60.20	1.05197	60.28

Standard uncertainties: u $\left( {m_{\text{A}} } \right)$ = ≤ 5.86 × $10^{ - 3}$ ${\text{mol}}{\cdot}{\text{kg}}^{ - 1}$, ${\text{u}}\left( {\phi_{{v}} } \right)$ ≤ 1.16 × $10^{ - 6} ( {\text{m}}^{3} {\cdot}{\text{mol}}^{ - 1} )$, ${\text{u}}\left( T \right)$ = 0.03 K, ${\text{u}}\left( p \right)$ = 0.5 kPa

**Fig. 5:**
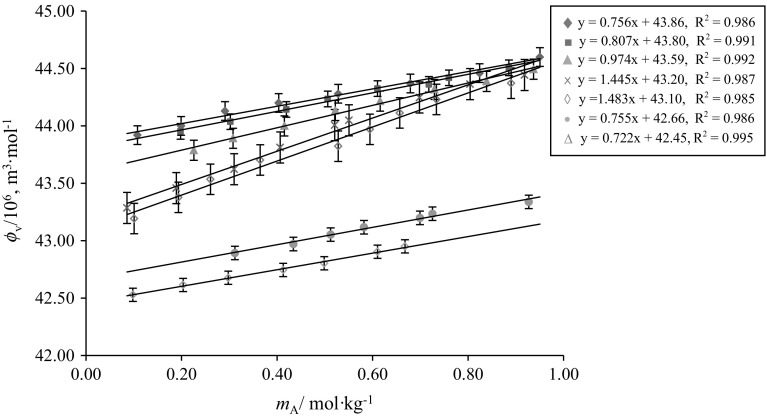
Representative plots of apparent molar volumes $\left( {\phi_{V} } \right)$ versus molality $\left( {m_{\text{A}} } \right)$ of glycine in water and in different concentrations of aqueous CA solutions at 288.15 K: (*m*_c_) = blue filled diamond, water; red filled square, 0.05; Green filled triangle, 0.10; Violet multiply symbol, 0.20; blue open diamond, 0.30; orange filled circle, 0.40; blue open triangle, 0.50 ${\text{mol}}{\cdot}{\text{kg}}^{ - 1}$ (Color figure online)

**Fig. 6:**
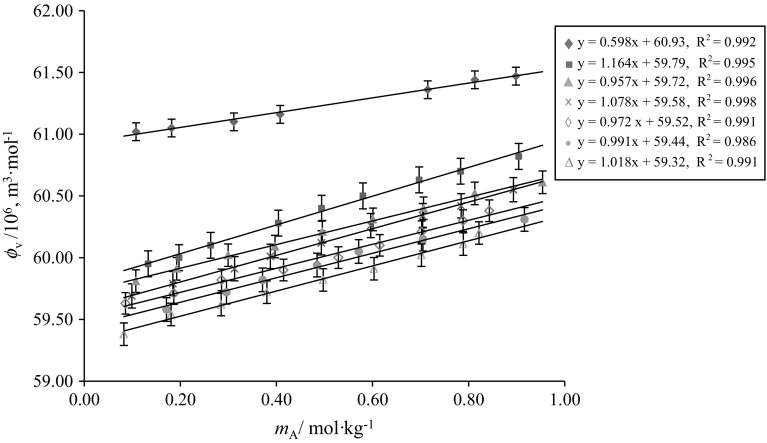
Representative plots of apparent molar volumes $\left( {\phi_{{v}} } \right)$ versus molality $\left( {m_{\text{A}} } \right)$ of l-alanine in water and in different concentrations of aqueous CA solutions at 288.15 K: $\left( {m_{\text{c}} } \right)$ = blue filled diamond, water; red filled square, 0.05; Green filled triangle, 0.10; Violet multiply symbol, 0.20; blue open diamond, 0.30; orange filled circle, 0.40; blue open triangle, 0.50 ${\text{mol}} {\cdot} {\text{kg}}^{ - 1}$ (Color figure online)

**Fig. 7:**
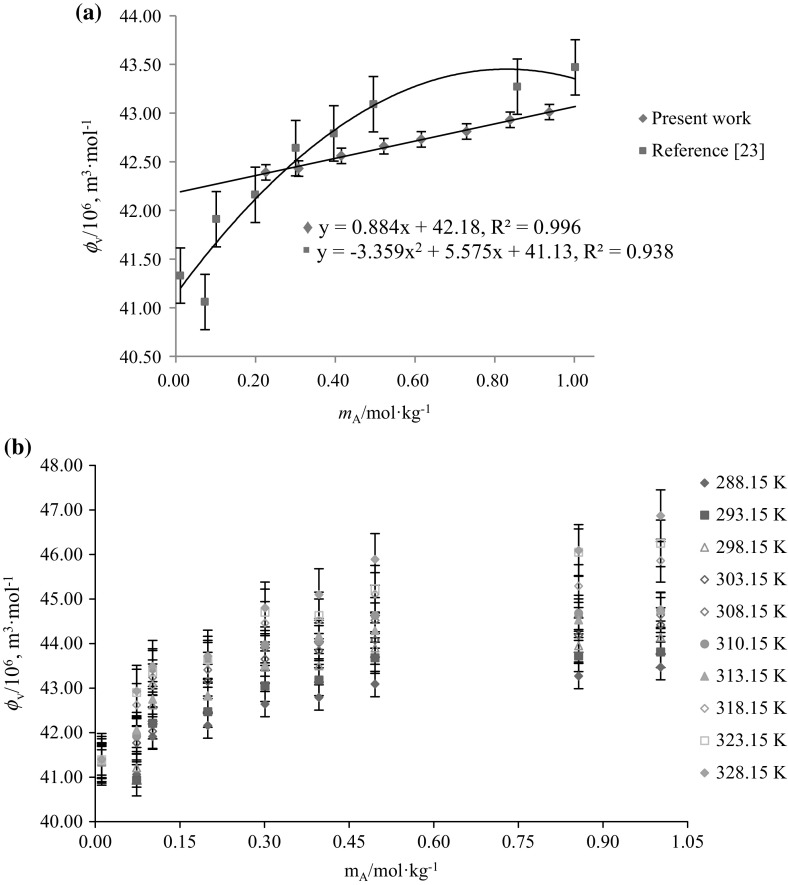
(**a**) Representative plots of apparent molar volumes $\left( {\phi_{V} } \right)$ versus molality $\left( {m_{\text{A}} } \right)$ of glycine in: blue filled diamond, 0.10 ${\text{mol}} {\cdot} {\text{kg}}^{ - 1}$ aqueous CA solutions and red filled square, 0.10 ${\text{mol}} {\cdot} {\text{kg}}^{ - 1}$ in aqueous succinic acid (abbreviated as SA) solutions at 288.15 K. **b** Plots of apparent molar volumes $\left( {\phi_{V} } \right)$ of glycine versus molality $\left( {m_{\text{A}} } \right)$ in 0.10 ${\text{mol}} {\cdot} {\text{kg}}^{ - 1}$ aqueous succinic acid solutions (abbreviated as SA) at different temperatures, *T* = (288.15–328.15) K (Color figure online)

**Scheme 1:**
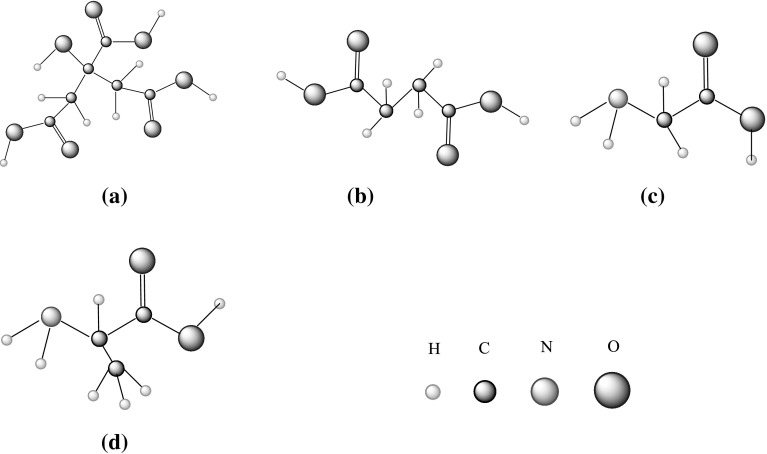
Basic structures of (**a**) citric acid, (**b**) succinic acid, (**c**) glycine and (**d**) l-alanine

The variation of $\phi_{V}$ versus $m_{\text{A}}$ for glycine/l-alanine in water and in different concentrations of aqueous CA solutions at 288.15 K are shown in Figs. 5 and 6, respectively (representative plots only). It is observed from Figs. 5 and 6 that the $\phi_{V}$ values of glycine/l-alanine in aqueous CA solutions vary almost linearly with increases in AA concentration as well as with temperature, whereas these values decrease with increases in the concentration of aqueous CA in these solutions. The higher $\phi_{V}$ values obtained for glycine/l-alanine in water indicate that strong solute–solvent interactions exist between glycine/l-alanine and water. In fact, strong interactions of the three carboxyl groups and one hydroxyl group of CA with water via hydrogen bonds leads to the higher $\phi_{V}$ values, however the hydrophobic group in SA [23] reduces its ability to form strong hydrogen bonds with water, thus resulting in smaller $\phi_{V}$ values (Fig. 7a). The basic structures of citric acid (CA), succinic acid (SA), glycine and l-alanine are given in Scheme 1.

**Fig. 8:**
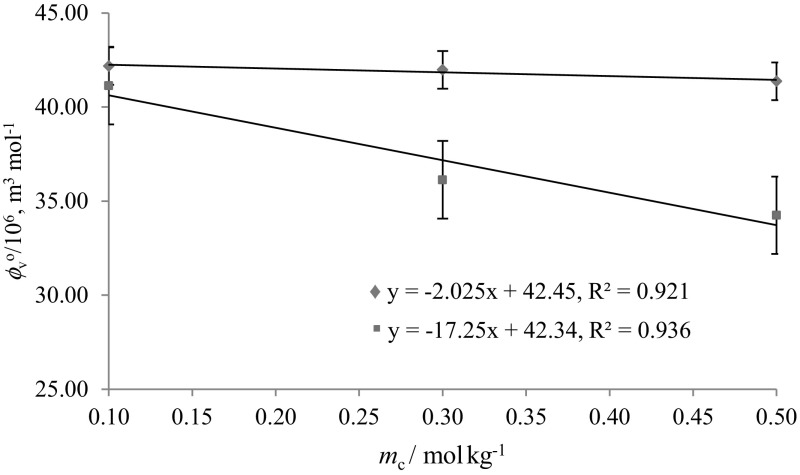
Representative plots of limiting partial molar volumes $\phi_V^o$ versus molality $\left( {m_{\text{c}} } \right)$ of glycine in: blue filled diamond, aqueous CA solutions and red filled square, in aqueous SA solutions at 288.15 K (Color figure online)

**Fig. 9:**
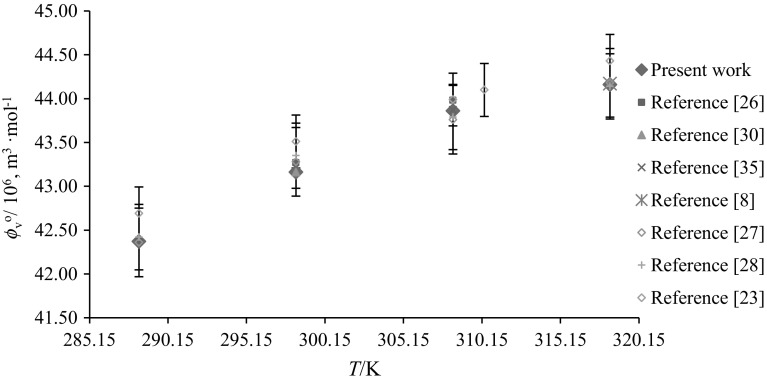
Comparative plots of limiting partial molar volumes $\left( {\phi_{V}^{\text{o}} } \right)$ of glycine in water versus temperature, *T* = (288.15–318.15) K

Solute–solute interactions are eliminated at infinite dilution and hence the apparent molar volume $(\phi_{V} )$ becomes equal to the limiting partial molar volume $(\phi_{V}^{\text{o}} )$. The $\phi_{V}^{\text{o}}$ values have been evaluated by least-squares fitting of the following equation to the corresponding data:

2 $$\phi_{V} = \phi_{V}^{\text{o}} + S_{V} m_{\text{A}}$$

where $\phi_{V}^{\text{o}}$ provides a measure of solute–solvent interactions, and the experimental slope $S_{\text{v}}$ provides information regarding solute–solute interactions [28]. $\phi_{V}^{\text{o}}$ and $S_{V}$ values of glycine/l-alanine in water and in aqueous CA solutions at different temperatures are listed in Table 3. The standard uncertainty of the limiting partial molar volumes has been found to be ≤ 0.07 × 10^−6^ m^3^·mol^−1^. The $\phi_{V}^{\text{o}}$ values of glycine/l-alanine in water agree well with the literature values at different temperatures and are shown in Figs. 8 and 9 [8, 23, 26–28, 30, 35, 36]. Table 3 shows that the $\phi_{V}^{\text{o}}$ values are higher for l-alanine in water and in aqueous CA solutions compared to glycine, as expected, which is due to the increase in the molar mass of l-alanine. The magnitude of the $\phi_{V}^{\text{o}}$ values is higher than the *S*_v_ values, which suggests that the extent of solute–solvent interactions is greater compared to solute–solute interactions. Moreover, the $\phi_{V}^{\text{o}}$ values decrease with increase in aqueous CA concentration whereas they increase with increasing temperature. The decrease in $\phi_{V}^{\text{o}}$ values with increasing concentration of CA may be attributed to the disruption of side group hydration by that of the charged end groups (a similar explanation has been given by Wang et al. [37], which supports our results for the $\phi_{V}^{\text{o}}$ values), whereas the increase in $\phi_{V}^{\text{o}}$ values of glycine/l-alanine in CA solutions with increase in temperature may lead to reduction of the electrostriction around the zwitterions [38]. Also, at higher temperatures, solvent from the secondary solvation layer of glycine/l-alanine is released into the bulk of solvent which results in the expansion of the solution and leads to higher $\phi_{V}^{\text{o}}$ values [39].

**Table 3:** Limiting partial molar volumes $\left( {\phi _{V}^{{\text{o}}} } \right)$ of glycine/l-alanine in water and in aqueous CA solutions at *T* = (288.15, 298.15, 308.15, 310.15 and 318.15) K

$m_{\text{c}}$ (mol·kg^−1^)	$10^{6} \times \phi _{V}^{{\text{o}}} \,({\text{m}}^{3} {\cdot}{\text{mol}}^{{ - 1}} )$				
	288.15, *T* (K)	298.15, *T* (K)	308.15, *T* (K)	310.15, *T* (K)	318.15, *T* (K)
Glycine					
0.00	42.37^a^ ± 0.02 (1.01)^b^ [0.99]^c^ 42.69^d^,42.37^e^, 42.38^f^	43.16^a^ ± 0.03 (0.93) [0.99] 43.51^d^, 43.27^e^, 42.28^f^ 43.16^g^,	43.86^a^ ± 0.02 (0.66) [0.99] 43.77^d^, 43.98^e^, 43.76^f^ 43.87^h^	43.87^a^ ± 0.03 (0.76) [0.99] 44.10^d^	44.16^a^ ± 0.02 (0.75) [0.99] 44.43^d^, 44.16^f^ 44.17^i^, 44.14^j^
0.05	42.32 ± 0.01 (0.92) [0.99]	43.14 ± 0.02 (0.92) [0.99]	43.61 ± 0.03 (0.96) [0.99]	43.80 ± 0.02 (0.81) [0.99]	44.10 ± 0.02 (0.82) [0.99]
0.10	42.18 ± 0.01 (0.82) [0.99]	43.07 ± 0.02 (0.89) [0.99]	43.54 ± 0.02 (0.95) [0.98]	43.60 ± 0.02 (0.97) [0.99]	43.87 ± 0.03 (1.02) [0.99]
0.20	42.08 ± 0.03 [0.99] [0.99]	42.69 ± 0.05 (1.34) [0.99]	43.16 ± 0.04 (1.39) [0.99]	43.20 ± 0.05 (1.44) [0.99]	43.35 ± 0.07 (1.59) [0.99]
0.30	41.98 ± 0.02 (0.92) [0.99]	42.46 ± 0.04 (1.48) [0.99]	42.89 ± 0.05 (1.66) [0.99]	43.10 ± 0.05 (1.48) [0.99]	43.29 ± 0.04 (1.65) [0.99]
0.40	41.43 ± 0.03 (1.35) [0.99]	41.82 ± 0.02 (1.04) [0.99]	42.31 ± 0.01 (0.54) [0.99]	42.66 ± 0.02 (0.77) [0.99]	42.97 ± 0.03 (0.74) [0.99]
0.50	41.37 ± 0.01 (1.18) [0.99]	41.63 ± 0.02 (1.72) [0.99]	42.26 ± 0.02 (0.73) [0.99]	42.46 ± 0.01 (0.72) [0.99]	42.94 ± 0.02 (1.42) [0.99]
l-Alanine					
0.00	59.66^a^ ± 0.02 (0.76) [0.99] 59.67^e^	60.35^a^ ± 0.02 (0.64) [0.99] 60.42^e^	60.84^a^ ± 0.02 (0.62) [0.99] 60.88^e^	60.94^a^ ± 0.02 (0.59) [0.99]	61.15^a^ ± 0.03 (0.74) [0.99]
0.05	59.53 ± 0.02 (0.97) [0.99]	59.62 ± 0.02 (1.19) [0.99]	59.71 ± 0.02 (1.18) [0.99]	59.80 ± 0.02 (1.16) [0.99]	60.00 ± 0.02 (1.04) [0.99]
0.10	59.44 ± 0.01 (0.96) [0.99]	59.51 ± 0.02 (0.98) [0.99]	59.62 ± 0.03 (1.01) [0.99]	59.72 ± 0.02 (0.96) [0.99]	59.85 ± 0.03 (1.09) [0.99]
0.20	59.31 ± 0.02 (1.01) [0.99]	59.48 ± 0.01 (0.94) [0.99]	59.57 ± 0.01 (0.95) [0.99]	59.59 ± 0.01 (1.08) [0.99]	59.74 ± 0.03 (1.14) [0.99]
0.30	59.21 ± 0.01 (0.90) [0.99]	59.31 ± 0.02 (1.02) [0.99]	59.42 ± 0.02 (0.95) [0.99]	59.52 ± 0.03 (0.97) [0.99]	59.61 ± 0.03 (1.05) [0.99]
0.40	59.13 ± 0.02 (0.92) [0.99]	59.26 ± 0.02 (0.94) [0.99]	59.36 ± 0.03 (0.98) [0.99]	59.44 ± 0.03 (0.99) [0.99]	59.52 ± 0.03 (1.09) [0.99]
0.50	59.05 ± 0.01 (1.00) [0.99]	59.12 ± 0.02 (1.08) [0.99]	59.28 ± 0.02 (0.95) [0.99]	59.32 ± 0.03 (1.02) [0.99]	59.44 ± 0.03 (1.00) [0.99]

^a^Present work

^b^$S_{\text{v}}$ $\times$ 10^6^ $\left( {{\text{m}}^{3} {\cdot} {\text{kg}} {\cdot} {\text{mol}}^{ - 2} } \right)$ values in parenthesis ()

^c^Regression coefficient values in square brackets []

^d^Reference [23]

^e^Reference [26]

^f^Reference [8]

^g^Reference [27]

^h^Reference [35]

^i^Reference [36]

^j^Reference [28]

The standard uncertainties in molality, u $\left( {m_{\text{A}} } \right)$ is ≤ 5.86 $\times10^{ - 3}$ ${\text{mol}} {\cdot} {\text{kg}}^{ - 1}$

The standard uncertainty in temperature, ${\text{u}}\left( T \right)$ is 0.03 K

The standard uncertainty in pressure, ${\text{u}}\left( p \right)$ is 0.5 kPa

The standard uncertainty in partial molar volume, *u* $\left( {\phi_{\text{v}}^{\text{o}} } \right)$ is ≤ 0.07 × $10^{ - 6} {\text{m}}^{3} {\cdot}{\text{mol}}^{ - 1}$

± Respective errors in $\phi_{V}^{\text{o}}$ values

**Fig. 10:**
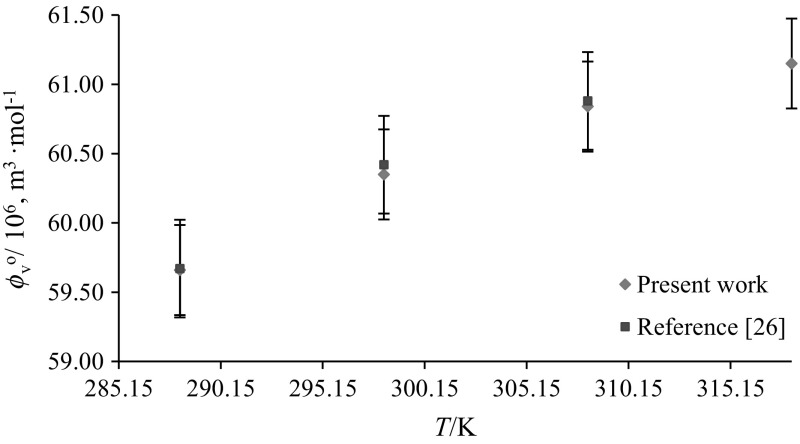
Comparative plots of limiting partial molar volumes $\left( {\phi_{V}^{\text{o}} } \right)$ of l-alanine in water versus temperature, *T* = (288.15–318.15) K

**Scheme 2:**
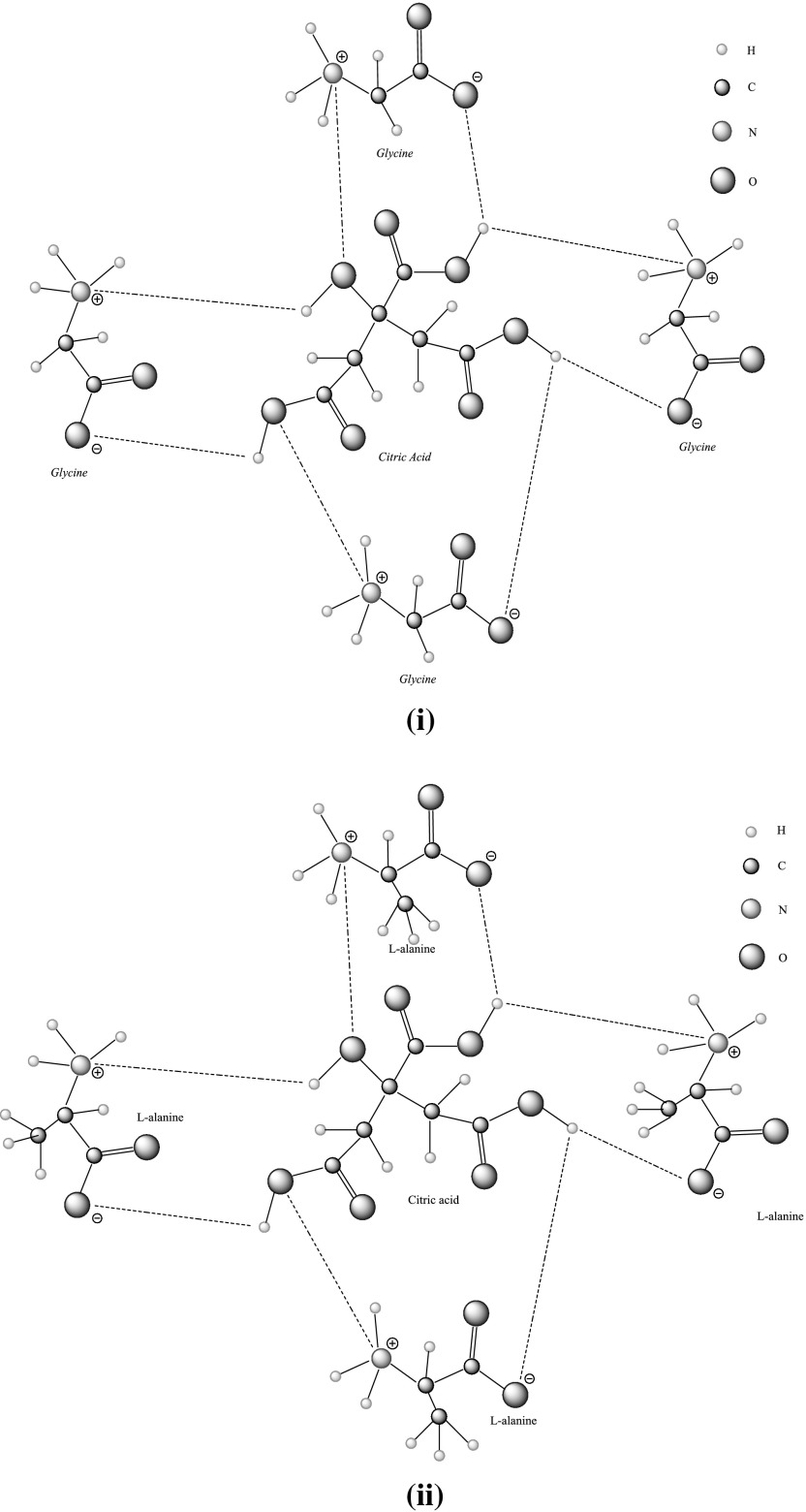
Different types of possible interactions (**i**) glycine–CA and (**ii**) l-alanine–CA at pH 2.12 and at *T* = 310.15 K

Comparison of $\phi_{V}$ and $\phi_{V}^{\text{o}}$ values of glycine in CA (present work) with previously reported data of glycine in SA [23] reveals that the magnitudes of $\phi_{V}$ and $\phi_{V}^{\text{o}}$ values for glycine–CA are higher than for glycine–SA (Figs. 7(a), (b), 10). As discussed earlier, the higher magnitudes for glycine in CA solutions are again attributed to the presence of additional hydrophilic groups (–OH and –COOH in CA) which leads to stronger interactions between glycine/l-alanine and CA, *i.e.* it is due to the formation of hydrogen bonds (Scheme 2) [40]. Further, the effect of pH on speciation and charge distribution of these systems involves the stronger interaction among citrate ion and the ionized ammonium group. The amino and carboxyl groups of glycine/l-alanine dissociate in aqueous citric acid solutions and form negatively and positively charged ions (*i.e.*, ^+^NH_3_-(CH(H/CH_3_)-COO^–^). Dissociation of citric acid in aqueous solutions [19], *i.e.* the negatively and positively charged ions, results in the formation of new species in aqueous solutions as:

$$^{ } {\text{NH}}_{ 3} {\text{RCOO}}^{-} \rightleftharpoons {\text{NH}}_{ 2} {\text{RCOO}}^{-} + {\text{ H}}^{ + }$$

$${\text{H}}_{ 3} {\text{Cit}}^{-} \rightleftharpoons {\text{H}}_{ 2} {\text{Cit}}^{-} + {\text{ H}}^{ + }$$

$${\text{H}}_{ 2} {\text{Cit}}^{-} \rightleftharpoons {\text{H}}^{ + } + {\text{ HCit}}^{{ 2{-}}}$$

$${\text{H}}_{ 2} {\text{O}} \rightleftharpoons {\text{H}}^{ + } + {\text{ OH}}^{-}$$

Partial molar volumes of transfer $(\Delta_{\text{tr}} \phi_{V} )$ of glycine/l-alanine from water to aqueous CA solutions at infinite dilution have been calculated by using the following equation:

3 $$\Delta_{\text{tr}} \phi_{{v}} ({\text{water}} \to {\text{aqueous}}\;{\text{CA solutions}}) = \phi_{V}^{\text{o}} ({\text{in aqueous}}\;{\text{CA solutions}}) - \phi_{V}^{\text{o}} ({\text{in}}\;{\text{water}})$$

**Fig. 11:**
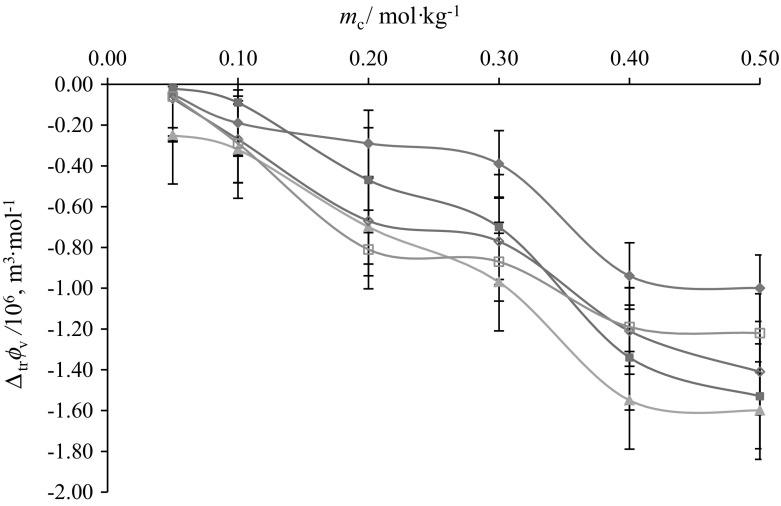
Plots of partial molar volumes of transfer $\left( {\Delta _{\text{tr}} \phi_{V} } \right)$ versus molalities $\left( {m_{\text{c}} } \right)$ of CA of glycine at different temperatures: *T* = blue filled diamond, 288.15, red filled square, 298.15; Green filled triangle, 308.15; Violet open diamond, 310.15; blue open square, 318.15 K (Color figure online)

**Fig. 12:**
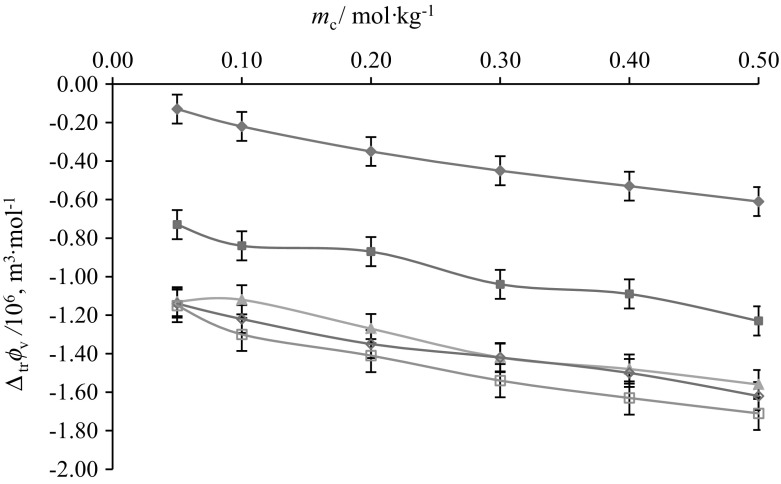
Plots of partial molar volumes of transfer $\left( {\Delta _{\text{tr}} \phi_{V} } \right)$ versus molalities $\left( {m_{c} } \right)$ of CA of l-alanine at different temperatures: *T *= blue filled diamond, 288.15; red filled square, 298.15; Green filled triangle, 308.15; Violet open diamond, 310.15; blue open square, 318.15 K (Color figure online)

The $\Delta_{\text{tr}} \phi_{{v}}$ values reported in Table S1 (supplementary material) are negative and decrease with increase in the concentration of aqueous CA solutions at the studied temperatures (Figs. 11 and 12). The standard uncertainty in $\Delta_{\text{tr}} \phi_{V}$ values has been found to be ≤ 0.07 × 10^−6^ m^3^·mol^−1^. The possible interactions which may exist between ternary system (*i.e.* glycine/l-alanine + aqueous CA) (Scheme 2) can be categorized as: (1) ion/hydrophilic–dipolar interactions between (3COO^–^, -OH) of CA and (NH_3_^+^, COO^–^) zwitterions of glycine/l-alanine, (2) hydrophilic–hydrophobic interactions between the (3COO^–^, -OH) groups of CA with the non-ionic group of glycine/l-alanine, (3) hydrophobic–hydrophilic interactions between the non-ionic group of CA and zwitterionic groups of glycine/l-alanine, and (4) hydrophobic–hydrophobic group interactions between the alkyl chains of glycine/l-alanine and CA. According to the co-sphere overlap model [41], the overlap of the hydration co-spheres of hydrophilic and ionic parts (type 1) results in positive $\Delta_{\text{tr}} \phi_{V}$ values, whereas interactions of type 2, 3 and 4 result in negative transfer volumes. The presently observed negative $\Delta_{\text{tr}} \phi_{V}$ values for glycine/l-alanine in aqueous CA solutions at different temperatures suggest the dominance of hydrophilic–hydrophobic and hydrophobic–hydrophobic interactions over ion/hydrophilic–dipolar interactions. The greater magnitude of $\Delta_{\text{tr}} \phi_{V}$ observed in the case of l-alanine in CA may be attributed to the presence of an alkyl group in l-alanine which further strengthens the view that hydrophobic–hydrophobic interactions are dominating in the case of l-alanine–CA (*i.e.* type 4 interactions).

The magnitude of $\phi_{V}^{\text{o}}$ values of glycine/l-alanine in CA can also be explained by considering the modified equation of Shahidi and Farrell [42],

4 $$\phi_{V}^{\text{o}} = V_{{v , {\text{w}}}} + V_{\text{void}} - V_{\text{shrinkage}}$$

where $V_{{v , {\text{w}}}}$ is the van der Waal’s volume, $V_{\text{void}}$ is the volume associated with voids, and $V_{\text{shrinkage}}$ is the volume due to shrinkage that arises from electrostriction of solvent molecules caused by hydrophilic groups present in the solute. Assuming that $V_{{v , {\text{w}}}}$ and $V_{\text{void}}$ are not significantly affected by the presence of CA, then the negative $\Delta_{\text{tr}} \phi_{V}$ values may be attributed to enhanced electrostriction in the vicinity of charged centers of zwitterions which results in an increase of $V_{\text{shrinkage}}$. Further, it is observed from Table S1 that the $\Delta_{\text{tr}} \phi_{V}$ values for glycine in CA are higher in contrast to glycine in SA solutions [23], which is attributed to the presence of additional hydrophilic groups in CA that leads to the formation of strong hydrogen bonding with glycine (Scheme 2).

The McMillan–Mayer theory of solutions [43, 44] permits the formal separation of the effects due to the interactions between two or more solutes. According to this theory, the pair and triplet interaction coefficients (*V*_AB_) and (*V*_ABB_) can be calculated from the partial molar volumes of transfer $(\Delta_{\text{tr}} \phi_{V} )$ by using the following equation:

5 $$\Delta_{\text{tr}} \phi_{V} = 2V_{\text{AB}} m_{\text{c}} + 3V_{\text{ABB}} m_{\text{c}}^{2} + \cdots$$

where A denotes glycine/l-alanine, B denotes CA and $m_{\text{c}}$ is the molality of CA. The *V*_AB_ and *V*_ABB_ values of glycine/l-alanine in aqueous CA solutions are given in Table 4. The *V*_AB_ values for glycine (except at 298.15 K) and l-alanine in aqueous CA solutions are found to be negative at different temperatures. On the other hand, the *V*_ABB_ values are negative for glycine and positive for l-alanine over the entire temperature range. From Table 4 it is also observed that the magnitude of *V*_AB_ and *V*_ABB_ values for l-alanine are greater than for glycine in CA, which suggest that l-alanine interacts more strongly with CA. Overall, the higher magnitude of *V*_ABB_ values for l-alanine in CA at the studied temperatures indicates the dominance of triplet interactions. Pair interactions dominate for glycine in the presence of CA at lower temperatures (*i.e.* 288.15 and 298.15 K) whereas at higher temperatures (*i.e.* 308.15, 310.15 and 318.15 K) triplet interactions dominate. The reverse trend of *V*_AB_ and *V*_ABB_ values has been observed for glycine in SA, *i.e. V*_AB_ dominates at high temperature whereas *V*_ABB_ dominates at low temperature (no specific reason mentioned). The observed behavior of *V*_AB_ and *V*_ABB_ values for glycine in CA may be attributed to the presence of some cooperativity in the interaction of the alkyl group (hydrocarbon part), *i.e.* when two hydrocarbon groups come in contact with each other then it is easier for the third group to join the other two [45].

**Table 4:** Pair $\left( {V_{\text{AB}} } \right)$ and triplet $\left( {V_{\text{ABB}} } \right)$ interaction coefficients of glycine/l-alanine in aqueous CA solutions at *T* = (288.15 to 318.15) K

*T*(K)	$10^{6} \times V_{{{\text{AB}}}} \,({\text{m}}^{3}{ \cdot}{\text{mol}}^{{ - 2}} {\cdot}{\text{kg)}}$	$10^{6} \times V_{{{\text{ABB}}}}$ $({\text{m}}^{3} {\cdot}{\text{mol}}^{ - 3} {\cdot}{\text{kg}}^{2} )$
Glycine		
288.15	− 0.33 (− 12.28)_SA_	− 1.62 (12.69)_SA_
298.15	0.19 (− 11.68)_SA_	− 5.07 (3.82)_SA_
308.15	− 1.40 (0.64)_SA_	− 1.69 (− 33.95)_SA_
310.15	− 1.40 (0.91)_SA_	− 0.22 (− 35.34)_SA_
318.15	− 1.64 (1.22)_SA_	− 0.59 (− 39.19)_SA_
l-Alanine		
288.15	− 1.32	1.75
298.15	− 5.82	13.51
308.15	− 8.35	19.42
310.15	− 8.99	21.48
318.15	− 9.25	21.58

()_SA_ are the $V_{\text{AB}}$ and $V_{\text{ABB}}$ values of glycine in aqueous SA solutions [23]

To study the effect of temperature on $\phi_{V}^{\text{o}}$, the limiting partial molar expansibilities $(\partial \phi_{V}^{\text{o}} /\partial T)_{p}$ and their second-order derivatives $(\partial^{2} \phi_{V}^{\text{o}} /\partial T^{2} )_{p}$ have been calculated by fitting the following equation to the corresponding data:

6 $$\phi_{V}^{\text{o}} = a + bT + cT^{2}$$

where *a, b* and *c* are constants and *T* is the absolute temperature. The $(\partial \phi_{V}^{\text{o}} /\partial T)_{p}$ and $(\partial^{2} \phi_{V}^{\text{o}} /\partial T^{2} )_{p}$ values of glycine/l-alanine in water are (0.097 m^3^·mol^−1^·K^−1^, − 0.0023 m^3^·mol^−1^·K^−2^) and (0.079 m^3^·mol^−1^·K^−1^, − 0.0019 m^3^·mol^−1^·K^−2^), respectively, which agree well with the literature values [27, 28, 34] and are summarized in Table 5. The $(\partial^{2} \phi_{V}^{\text{o}} /\partial T^{2} )_{p}$ values of glycine in aqueous CA solutions decrease with increasing temperature except at $m_{{{\text{c}} }}$ = (0.4 and 0.5) mol·kg^−1^ whereas the $(\partial^{2} \phi_{V}^{\text{o}} /\partial T^{2} )_{p}$ values for l-alanine increase with increasing temperature.

**Table 5:** Limiting partial molar expansibilities $(\partial \phi _{V}^{{\text{o}}} /\partial T)_{p}$ and their second-order derivatives $(\partial ^{2} \phi _{V}^{{\text{o}}} /\partial T^{2} )_{p}$ for glycine/l-alanine in water and in aqueous CA solutions at *T* = (288.15 to 318.15) K

$m_{\text{c}}$ (mol·kg^−1^)	$(\partial \phi _{V}^{{\text{o}}} /\partial T)_{p}$ (m^3^·mol^−1^·K^−1^)					$(\partial ^{2} \phi _{V}^{{\text{o}}} /\partial T^{2} )_{p}$ (m^3^·mol^−1^·K^−2^)
	*T*(K): 288.15	298.15	308.15	310.15	318.15	
Glycine						
0.00	0.096^a^ (0.097)^b^	0.072^a^ (0.072)^b^ (0.071)^c^	0.049^a^ (0.047)^b^	0.044^a^	0.025^a^ (0.022)^b^	− 0.0024^a^ (− 0.0025)^b^
0.05	0.084	0.067	0.049	0.046	0.032	− 0.0018
0.10	0.097 (0.125)_SA_	0.071 (0.093)_SA_	0.044 (0.061)_SA_	0.039 (0.054)_SA_	0.018 (0.029)_SA_	− 0.0026 (− 0.0032)_SA_
0.20	0.075	0.054	0.033	0.029	0.012	− 0.0021
0.30	0.056 (0.486)_SA_	0.048 (0.416)_SA_	0.041 (0.346)_SA_	0.040 (0.332)_SA_	0.034 (0.276)_SA_	− 0.0007 (− 0.0074)_SA_
0.40	0.041	0.049	0.057	0.058	0.065	0.0008
0.50	0.027 (− 0.219)_SA_	0.045 (− 0.149)_SA_	0.063 (− 0.079)_SA_	0.067 (− 0.065)_SA_	0.081 (− 0.009)_SA_	0.0018 (0.0075)_SA_
l-Alanine						
0.00	0.079	0.060 (0.062)^d^	0.040	0.036	0.021	− 0.0019
0.05	0.008	0.010	0.020	0.022	0.029	− 0.0010
0.10	0.004	0.010	0.017	0.018	0.024	0.0007
0.20	0.012	0.013	0.014	0.014	0.015	0.0001
0.30	0.009	0.012	0.015	0.016	0.018	0.0003
0.40	0.010	0.013	0.015	0.015	0.017	0.0002
0.50	0.007	0.011	0.015	0.016	0.019	0.0004

()_SA_ are the $(\partial \phi_{V}^{\text{o}} /\partial T)_{p}$ and $(\partial^{2} \phi_{V}^{\text{o}} /\partial T^{2} )_{p}$ values of glycine in aqueous SA solutions [23]

^a^Present work

^b^Reference [27]

^c^Reference [28]

^d^Reference [34]

Hepler [46] used the following thermodynamic relation by which qualitative information regarding hydration of a solute can be evaluated from the thermal expansion:

7 $$(\partial C_{p}^{\text{o}} /\partial p)_{T} = - T(\partial^{2} \phi_{V}^{\text{o}} /\partial T^{2} )_{p}$$

where $C_{p}^{\text{o}}$ is the partial molar heat capacity. The sign of $(\partial C_{p}^{\text{o}} /\partial p)_{T}$ and its temperature dependence should provide a distinction between the structure making or breaking ability of solutes in solution. According to Eq. 7, a structure-breaking solute should have negative $(\partial^{2} \phi_{V}^{\text{o}} /\partial T^{2} )_{p}$ values whereas positive $(\partial^{2} \phi_{V}^{\text{o}} /\partial T^{2} )_{p}$ values suggest that the solute behaves as a structure maker. It can be seen from Table 5 that glycine predominantly acts as a structure breaker whereas l-alanine acts as a structure maker. It is concluded that the structure-breaking and structure-making behavior of glycine and l-alanine in CA may be attributed to the absence of the caging effect [47]. Similar behavior for glycine in SA is observed for the $(\partial^{2} \phi_{V}^{\text{o}} /\partial T^{2} )_{p}$ values. Overall, the structure breaking tendency of glycine in the presence of CA is higher than for glycine in SA [23]. This may be attributed due to partial dissociation of CA which tends to destruct the hydrogen bonded structure of water, and then water behaves as a normal (*i.e.* non associated) liquid. The equilibrium of different water species (*i.e.*, a hydrogen bonded structure associated with a normal liquid) is temperature dependent. Elevation in temperature leads to the expansion of volume with increase in the fraction of non-associated water molecules. In addition, the water structure changes from the combined effects of increased temperature and with increase in the CA concentration [19].

### Hydration Number

**Fig. 13:**
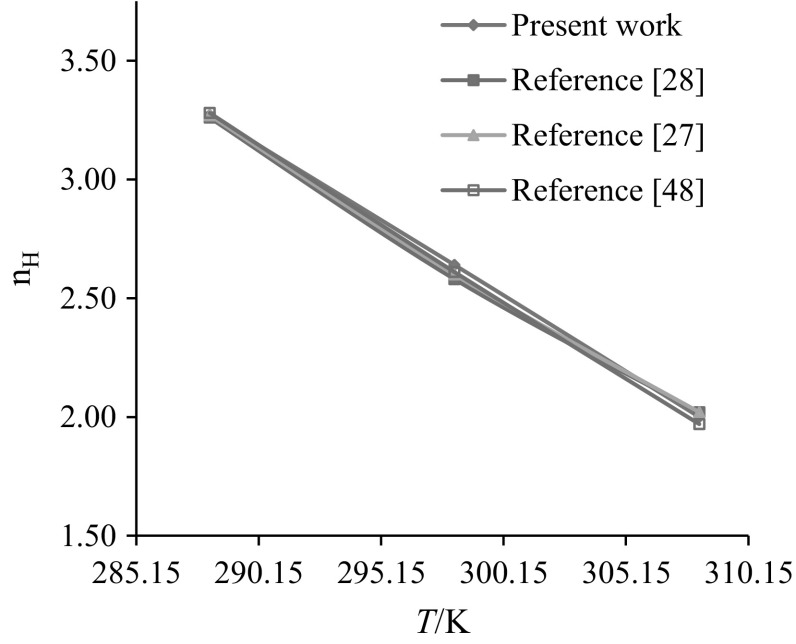
Comparison plots of hydration number ($n_{\text{H}} )$ of glycine in water at different temperatures, *T *= (288.15, 298.15 and 308.15) K

**Fig. 14:**
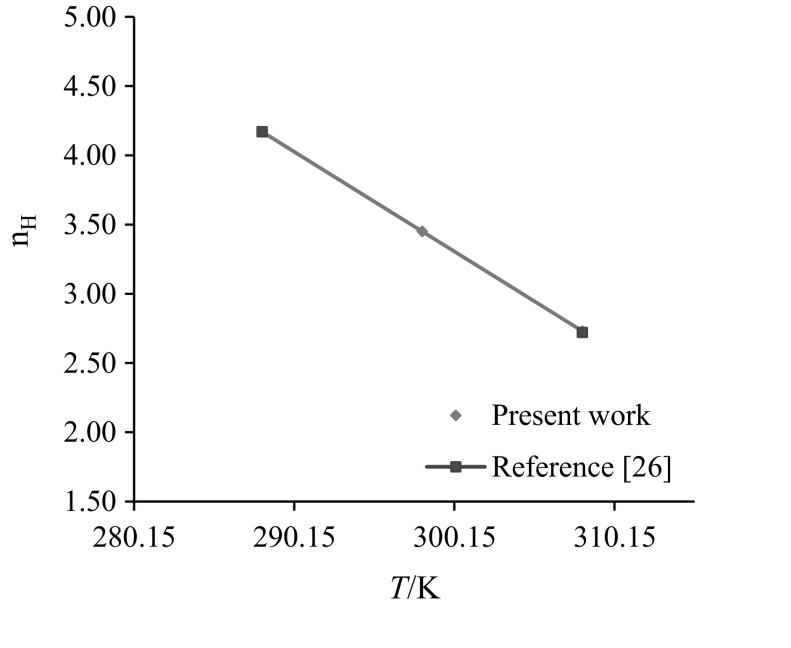
Comparison plots of hydration number ($n_{\text{H}} )$ of l-alanine in water at different temperatures, *T *= (288.15, 298.15 and 308.15) K

The hydration number $\left( {n_{\text{H}} } \right)$ reflects the electrostriction effect of the charge centers of amino acids on nearby water molecules. Millero et al. [48] reported a relationship between the limiting electrostriction contribution to the partial molar volume and hydration number of the non-electrolytes as:

8 $$\phi _{{V,\;{\text{elect}}}} = n_{{\text{H}}} (\phi _{{V,\;{\text{e}}}}^{{\text{o}}} - \phi _{{V,\;{\text{b}}}}^{{\text{o}}} )$$

where $\phi_{{V , {\text{e}}}}^{\text{o}}$ is the limiting molar volume of electrostricted water and $\phi_{{V , {\text{b}}}}^{\text{o}}$ is the molar volume of bulk water. For every water molecule taken from the bulk phase to the region near an AA, the $(\phi_{{V , {\text{e}}}}^{\text{o}} - \phi_{{V , {\text{b}}}}^{\text{o}} )$ values are − 2.9, − 3.3 and − 4.0) × 10^−2^ m^3^·mol^−1^ at *T* = (288.15, 298.15 and 308.15) K, respectively [48]. The $n_{\text{H}}$ values of glycine/l-alanine in water and in aqueous CA solutions are summarized in Table 6. The $n_{\text{H}}$ values of glycine/l-alanine in water are in good agreement with the literature values and are shown in Figs. 13 and 14 [26–28, 49]. The $n_{\text{H}}$ values of glycine and l-alanine increase with increase in the concentration of CA, which further suggests that water in the immediate vicinity of charged centers of glycine/l-alanine in aqueous CA solutions is highly electrostricted leading to higher $n_{\text{H}}$ values. The decrease in $n_{\text{H}}$ values with increases in temperature may be attributed to the weakening of the electrostriction effect of charged centers, which then leads to stronger interactions between glycine–CA and l-alanine–CA. It also shows that CA exerts a dehydration effect in the present systems.

**Table 6:** Hydration number $\left( {n_{\text{H}} } \right)$ of glycine/l-alanine in water and in aqueous CA solutions at *T* = (288.15, 298.15 and 308.15) K

*m*_c_ (mol·kg^−1^)	$n_{\text{H}}$		
	*T(*K): 288.15	298.15	308.15
Glycine			
0.00	3.27^a^ (3.26^b^, 3.27^c^, 3.28^d^)	2.64^a^ (2.58^b^, 2.60^c^, 2.61^d^)	2.00^a a^ (2.02^b,c^, 1.97^d^)
0.05	3.29	2.64	2.06
0.10	3.34 (3.97)_SA_	2.66 (3.20) _SA_	2.08 (2.55) _SA_
0.20	3.37	2.78	2.18
0.30	3.41 (5.83) _SA_	2.85 (4.37) _SA_	2.24 (2.38) _SA_
0.40	3.60	3.04	2.39
0.50	3.62 (6.53) _SA_	3.10 (5.27) _SA_	2.40 (4.32) _SA_
l-Alanine			
0.00	4.17^a^ (4.17^d^)	3.45^a^ (3.45^b^)	2.73^a^ (2.68^b^, 2.72^d^)
0.05	4.21	3.68	3.01
0.10	4.24	3.71	3.03
0.20	4.29	3.72	3.05
0.30	4.32	3.77	3.08
0.40	4.35	3.78	3.10
0.50	4.38	3.83	3.12

()_SA_ are the $n_{\text{H}}$ values of glycine in aqueous SA solutions [23]

^a^Present work

^b^Reference [28]

^c^Reference [27]

^d^Reference [49]

Lower $n_{\text{H}}$ values for glycine/l-alanine in water than in CA indicate that glycine/l-alanine are more hydrated in the presence of CA. Also, the higher $n_{\text{H}}$ values for glycine in SA than in CA further suggests that SA has a strong dehydrating effect on glycine. This may be due to the presence of hydrophilic groups (*i.e.*, –OH and –COOH in CA) which leads to the formation of hydrogen bonding.

### Apparent Specific Volumes and Taste Quality

CA and inorganic citrates are active ingredients in many dosage forms. It is a natural preservative which is used to add an acidic or sour taste to foods and drinks. The taste behavior can be verified on the basis of the apparent specific volumes ($v_{\phi }$) which gives a direct measure of the dislocation of water molecule by the solute and reflects its compatibility with water. $v_{\phi }$ has been calculated by using the following equation [50]:

9 $$v_{\phi } = \phi_{{v}} /M$$

where $\phi_{V}$ is the apparent molar volume and *M* is the molar mass of glycine/l-alanine and $v_{\phi } \times 10^{ - 3} {\text{m}}^{3} {\cdot} {\text{kg}}^{ - 1}$ bears a relationship to taste quality in the order salt < ∼ 0.33, sour ∼ 0.33 to ~ 0.52, sweet ∼ 0.52 to ~ 0.71, and bitter ∼ 0.71 to ∼ 0.93 [51]. The $v_{\phi }$ values for CA in water, glycine/l-alanine in water, in aqueous CA and glycine in aqueous SA solutions are given in Table S2. It is observed from Table S2 that the $v_{\phi }$ values of glycine/l-alanine in water and in aqueous CA solutions (range from 0.55 to 0.69) fall in the sweet taste behavior range (except for l-alanine at $m_{\text{c}}$ = 0.05 ${\text{mol}} {\cdot} {\text{kg}}^{ - 1}$), which may be due to hydrophobic interactions occurring in these ternary systems. The result obtained from the $v_{\phi }$ values also supports $\Delta _{\text{tr}} \phi_{V}$ data, which further strengthens the view that hydrophobic interactions are dominating in these systems. In the case of SA, the $v_{\phi }$ values of glycine tend to show sour-to-sweet taste behavior (ranges from 0.47 to 0.61) with increase in concentration and temperature. The obtained trend in SA may due to the displacement of a large number of water molecules by hydrophobic groups [52].

## Conclusions

The negative $\Delta_{\text{tr}} \phi_{V}$ values obtained for glycine/l-alanine in aqueous CA solutions suggest the dominance of hydrophilic–hydrophobic and hydrophobic–hydrophobic interactions in these systems. Triplet interactions dominate over pair interactions in the case of l-alanine compared to glycine in CA, which indicates that l-alanine interacts more strongly with CA. At low concentrations of CA, glycine/l-alanine act as structure breakers whereas at high concentrations of CA, glycine/l-alanine act as structure makers. Also, it is evident that CA has a dehydration effect on amino acids. The results obtained for $\upsilon_{\phi}$ values suggest that CA enhances the sweet taste behavior of glycine/l-alanine with rise in temperatures. Comparative studies of glycine in aqueous CA and aqueous SA solutions show that the stronger interactions exist between glycine–CA than for glycine–SA, which is attributed due to the presence of additional hydrophilic groups in CA that leads to the formation of hydrogen bonds.

## Electronic supplementary material

Below is the link to the electronic supplementary material.

Supplementary material 1 (DOCX 61 kb)
