# Supplementary material for: Volumetric Investigations on Molecular Interactions of Glycine/l-alanine in Aqueous Citric Acid Solutions at Different Temperatures
Source: J Solution Chem. 2018 Nov 19;47(12):2039–67. doi: 10.1007/s10953-018-0829-6 (PMC6267150; doi:10.1007/s10953-018-0829-6)
Supplement: Supplementary file 1 — Supplementary material 1 (DOCX 61 kb) [file 10953_2018_829_MOESM1_ESM.docx]

**Table S1** Partial molar volumes of transfer $\left( \Delta_{\mathrm{tr}}\phi_{V} \right)$of glycine/L-alanine from water to aqueous CA solutions at *T* = (288.15, 298.15, 308.15, 310.15 and 318.15) K

|  | $\Delta_{\mathrm{tr}}\phi_{V}{10}^{6} m^{3}\cdot\mathrm{mol}^{-1}$ | | | | | | | |
| --- | --- | --- | --- | --- | --- | --- | --- | --- |
| *T*/K: | 288.15 | 298.15 | 308.15 | | 310.15 | | 318.15 | |
| $m_{c}{/mol\cdot\mathrm{kg}}^{-1}$  ↓ | Glycine | | | | | | | |
| 0.05 | –0.05±0.02 | –0.02±0.04 | –0.25±0.04 | | –0.07±0.04 | | –0.06±0.03 | |
| 0.10 | –0.19±0.02  (–1.55)_SA_ | –0.09±0.04  (–2.21)_SA_ | –0.32±0.03  (–2.34)_SA_ | | –0.27±0.04  (–2.38)_SA_ | | –0.29±0.04  (–2.64) _SA_ | |
| 0.20 | –0.29±0.04 | –0.47±0.06 | –0.70±0.04 | | –0.67±0.06 | | –0.81±0.07 | |
| 0.30 | –0.39±0.03  (–6.56)_SA_ | –0.70±0.05  (–6.07) _SA_ | –0.97±0.05  (–1.66) _SA_ | | –0.77±0.06  (–1.55) _SA_ | | –0.87±0.04  (–1.31) _SA_ | |
| 0.40 | –0.94±0.04 | –1.34±0.04 | –1.55±0.02 | | –1.21±0.04 | | –1.19±0.04 | |
| 0.50 | –1.00±0.02  (–8.45) _SA_ | –1.53±0.04  (–9.03) _SA_ | –1.60±0.03  (–9.39) _SA_ | | –1.41±0.03  (–9.52) _SA_ | | –1.22±0.03  (–9.58) _SA_ | |
|  | L-alanine | | | | | | | |
| 0.05 | –0.13±0.09 | –0.73±0.09 | | –1.13±0.09 | | –1.14±0.09 | | –1.15±0.04 |
| 0.10 | –0.22±0.02 | –0.84±0.09 | | –1.12±0.04 | | –1.22±0.09 | | –1.30±0.04 |
| 0.20 | –0.35±0.09 | –0.87±0.02 | | –1.27±0.02 | | –1.35±0.02 | | –1.41±0.04 |
| 0.30 | –0.45±0.02 | –1.04±0.09 | | –1.42±0.09 | | –1.42±0.04 | | –1.54±0.04 |
| 0.40 | –0.53±0.09 | –1.09±0.09 | | –1.48±0.04 | | –1.50±0.04 | | –1.63±0.04 |
| 0.50 | –0.61±0.02 | –1.23±0.09 | | –1.56±0.09 | | –1.62±0.04 | | –1.71±0.04 |

$m_{c}$ is the molality of CA.

± are the respective errors in $\mathrm{the} \Delta_{\mathrm{tr}}\phi_{V}$ values.

( )_SA_ are the $\Delta_{\mathrm{tr}}\phi_{V}$ values of glycine in aqueous SA solutions [23].

The standard uncertainty in $\Delta_{\mathrm{tr}}\phi_{V}$ values is ≤ 0.07${10}^{-6} m^{3}\cdot\mathrm{mol}^{-1}$.

**Table S2** Taste behavior of glycine/L-alanine in water, in aqueous CA solutions and glycine in aqueous SA solutions at *T* = (288.15, 298.15, 308.15, 310.15 and 318.15) K

| $m_{A}$  $\left( {\mathrm{mol}\cdot\mathrm{kg}}^{-1} \right)$  ↓ | $v_{\phi}{10}^{-3} m^{3}{\cdot\mathrm{kg}}^{-1}$ | | | | |
| --- | --- | --- | --- | --- | --- |
|  | *T* (K): 288.15 | 298.15 | 308.15 | 310.15 | 318.15 |
|  | Glycine in water | | | | |
| 0.0000 | 0.56 | 0.58 | 0.58 | 0.58 | 0.59 |
| 0.1078 | 0.57 | 0.58 | 0.58 | 0.59 | 0.59 |
| 0.1991 | 0.57 | 0.58 | 0.59 | 0.59 | 0.59 |
| 0.2911 | 0.57 | 0.58 | 0.59 | 0.59 | 0.59 |
| 0.4034 | 0.57 | 0.58 | 0.59 | 0.59 | 0.59 |
| 0.5282 | 0.57 | 0.58 | 0.59 | 0.59 | 0.59 |
| 0.6791 | 0.57 | 0.58 | 0.59 | 0.59 | 0.59 |
| 0.8244 | 0.58 | 0.58 | 0.59 | 0.59 | 0.60 |
| 0.9504 | 0.58 | 0.59 | 0.59 | 0.59 | 0.60 |
| Glycine in 0.05 $\left( {\mathrm{mol}\cdot\mathrm{kg}}^{-1} \right)$ CA | | | | | |
| 0.0000 | 0.66 | 0.67 | 0.68 | 0.68 | 0.68 |
| 0.1981 | 0.57 | 0.58 | 0.58 | 0.59 | 0.59 |
| 0.3022 | 0.57 | 0.58 | 0.58 | 0.59 | 0.59 |
| 0.4196 | 0.57 | 0.58 | 0.59 | 0.59 | 0.59 |
| 0.5062 | 0.57 | 0.58 | 0.59 | 0.59 | 0.59 |
| 0.6103 | 0.57 | 0.58 | 0.59 | 0.59 | 0.59 |
| 0.7179 | 0.57 | 0.58 | 0.59 | 0.59 | 0.60 |
| 0.7599 | 0.57 | 0.58 | 0.59 | 0.59 | 0.60 |
| 0.8538 | 0.57 | 0.59 | 0.59 | 0.59 | 0.60 |
| Glycine in 0.10 $\left( {\mathrm{mol}\cdot\mathrm{kg}}^{-1} \right)$ CA | | | | | |
| 0.0000 | 0.57 | 0.58 | 0.59 | 0.59 | 0.60 |
| 0.2258 | 0.56 | 0.58 | 0.58 | 0.58 | 0.59 |
| 0.3080 | 0.57 | 0.58 | 0.58 | 0.58 | 0.59 |
| 0.4154 | 0.57 | 0.58 | 0.59 | 0.59 | 0.59 |
| 0.5221 | 0.57 | 0.58 | 0.59 | 0.59 | 0.59 |
| 0.6159 | 0.57 | 0.58 | 0.59 | 0.59 | 0.59 |
| 0.7295 | 0.57 | 0.58 | 0.59 | 0.59 | 0.59 |
| 0.8388 | 0.57 | 0.58 | 0.59 | 0.59 | 0.60 |
| 0.9371 | 0.57 | 0.58 | 0.59 | 0.59 | 0.60 |
| Glycine in 0.20 $\left( {\mathrm{mol}\cdot\mathrm{kg}}^{-1} \right)$ CA | | | | | |
| 0.0000 | 0.57 | 0.58 | 0.59 | 0.59 | 0.60 |
| 0.0859 | 0.56 | 0.57 | 0.58 | 0.58 | 0.58 |
| 0.1891 | 0.56 | 057 | 0.58 | 0.58 | 0.58 |
| 0.3102 | 0.57 | 0.58 | 0.58 | 0.58 | 0.58 |
| 0.4066 | 0.57 | 0.58 | 0.58 | 0.59 | 0.59 |
| 0.5207 | 0.57 | 0.58 | 0.59 | 0.59 | 0.59 |
| 0.5506 | 0.57 | 0.58 | 0.59 | 0.59 | 0.59 |
| 0.6985 | 0.57 | 0.58 | 0.59 | 0.59 | 0.59 |
| 0.8041 | 0.57 | 0.58 | 0.59 | 0.59 | 0.59 |
| 0.9182 | 0.57 | 0.58 | 0.59 | 0.59 | 0.60 |
| Glycine in 0.30 $\left( {\mathrm{mol}\cdot\mathrm{kg}}^{-1} \right)$ CA | | | | | |
| 0.0000 | 0.57 | 0.57 | 0.59 | 0.59 | 0.59 |
| 0.1008 | 0.56 | 0.57 | 0.57 | 0.58 | 0.58 |
| 0.1934 | 0.56 | 0.57 | 0.58 | 0.58 | 0.58 |
| 0.2606 | 0.56 | 0.57 | 0.58 | 0.58 | 0.58 |
| 0.3647 | 0.56 | 0.57 | 0.58 | 0.58 | 0.58 |
| 0.5281 | 0.57 | 0.58 | 0.58 | 0.58 | 0.58 |
| 0.5944 | 0.57 | 0.58 | 0.58 | 0.59 | 0.59 |
| 0.6569 | 0.57 | 0.58 | 0.59 | 0.59 | 0.59 |
| 0.7337 | 0.57 | 0.58 | 0.59 | 0.59 | 0.59 |
| 0.8901 | 0.57 | 0.58 | 0.59 | 0.59 | 0.60 |
| Glycine in 0.40 $\left( {\mathrm{mol}\cdot\mathrm{kg}}^{-1} \right)$ CA | | | | | |
| 0.0000 | 0.56 | 0.57 | 0.59 | 0.58 | 0.58 |
| 0.3118 | 0.56 | 0.56 | 0.57 | 0.57 | 0.58 |
| 0.4341 | 0.56 | 0.56 | 0.57 | 0.57 | 0.58 |
| 0.5122 | 0.56 | 0.56 | 0.57 | 0.57 | 0.58 |
| 0.5820 | 0.56 | 0.57 | 0.57 | 0.57 | 0.58 |
| 0.6992 | 0.56 | 0.57 | 0.57 | 0.58 | 0.58 |
| 0.7254 | 0.57 | 0.57 | 0.57 | 0.58 | 0.58 |
| 0.9271 | 0.57 | 0.57 | 0.57 | 0.58 | 0.58 |
| Glycine in 0.50 $\left( {\mathrm{mol}\cdot\mathrm{kg}}^{-1} \right)$ CA | | | | | |
| 0.0000 | 0.52 | 0.53 | 0.54 | 0.54 | 0.55 |
| 0.0981 | 0.55 | 0.56 | 0.56 | 0.57 | 0.57 |
| 0.2034 | 0.55 | 0.56 | 0.57 | 0.57 | 0.58 |
| 0.2978 | 0.56 | 0.56 | 0.57 | 0.57 | 0.58 |
| 0.4130 | 0.56 | 0.56 | 0.57 | 0.57 | 0.58 |
| 0.4988 | 0.56 | 0.57 | 0.57 | 0.57 | 0.58 |
| 0.6104 | 0.56 | 0.57 | 0.57 | 0.57 | 0.58 |
| 0.6678 | 0.56 | 0.57 | 0.57 | 0.57 | 0.58 |
| L-alanine in water | | | | | |
| 0.0000 | 0.67 | 0.68 | 0.68 | 0.68 | 0.69 |
| 0.1078 | 0.67 | 0.68 | 0.68 | 0.68 | 0.69 |
| 0.1818 | 0.67 | 0.68 | 0.68 | 0.69 | 0.69 |
| 0.3114 | 0.67 | 0.68 | 0.69 | 0.69 | 0.69 |
| 0.4077 | 0.67 | 0.68 | 0.69 | 0.69 | 0.69 |
| 0.7149 | 0.68 | 0.68 | 0.69 | 0.69 | 0.69 |
| 0.8130 | 0.68 | 0.68 | 0.69 | 0.69 | 0.69 |
| 0.8986 | 0.68 | 0.68 | 0.69 | 0.69 | 0.69 |
| L-alanine in 0.05 $\left( {\mathrm{mol}\cdot\mathrm{kg}}^{-1} \right)$ CA | | | | | |
| 0.0000 | 0.40 | 0.40 | 0.42 | 0.42 | 0.42 |
| 0.1330 | 0.67 | 0.67 | 0.67 | 0.67 | 0.67 |
| 0.1973 | 0.67 | 0.67 | 0.67 | 0.67 | 0.68 |
| 0.2631 | 0.67 | 0.67 | 0.67 | 0.67 | 0.68 |
| 0.4041 | 0.67 | 0.67 | 0.68 | 0.68 | 0.68 |
| 0.4940 | 0.67 | 0.68 | 0.68 | 0.68 | 0.68 |
| 0.5800 | 0.68 | 0.68 | 0.68 | 0.68 | 0.68 |
| 0.6971 | 0.68 | 0.68 | 0.68 | 0.68 | 0.68 |
| 0.7833 | 0.68 | 0.68 | 0.68 | 0.68 | 0.68 |
| 0.9042 | 0.68 | 0.68 | 0.68 | 0.68 | 0.68 |
| L-alanine in 0.10 $\left( {\mathrm{mol}\cdot\mathrm{kg}}^{-1} \right)$ CA | | | | | |
| 0.0000 | 0.44 | 0.45 | 0.46 | 0.46  Continued.. | 0.47 |
| 0.1073 | 0.67 | 0.67 | 0.67 | 0.67 | 0.67 |
| 0.1924 | 0.67 | 0.67 | 0.67 | 0.67 | 0.67 |
| 0.2994 | 0.67 | 0.67 | 0.67 | 0.67 | 0.68 |
| 0.3970 | 0.67 | 0.67 | 0.67 | 0.67 | 0.68 |
| 0.4955 | 0.67 | 0.67 | 0.67 | 0.68 | 0.68 |
| 0.6009 | 0.67 | 0.67 | 0.68 | 0.68 | 0.68 |
| 0.7062 | 0.67 | 0.68 | 0.68 | 0.68 | 0.68 |
| 0.8129 | 0.68 | 0.68 | 0.68 | 0.68 | 0.68 |
| 0.9539 | 0.68 | 0.68 | 0.68 | 0.68 | 0.68 |
| L-alanine in 0.20 $\left( {\mathrm{mol}\cdot\mathrm{kg}}^{-1} \right)$ CA | | | | | |
| 0.0000 | 0.50 | 0.51 | 0.52 | 0.52 | 0.53 |
| 0.0990 | 0.67 | 0.67 | 0.67 | 0.67 | 0.67 |
| 0.1844 | 0.67 | 0.67 | 0.67 | 0.67 | 0.68 |
| 0.3128 | 0.67 | 0.67 | 0.67 | 0.67 | 0.68 |
| 0.3873 | 0.67 | 0.67 | 0.67 | 0.67 | 0.68 |
| 0.4935 | 0.67 | 0.67 | 0.67 | 0.68 | 0.68 |
| 0.5962 | 0.67 | 0.67 | 0.68 | 0.68 | 0.68 |
| 0.7044 | 0.67 | 0.67 | 0.68 | 0.68 | 0.68 |
| 0.7844 | 0.67 | 0.68 | 0.68 | 0.68 | 0.68 |
| 0.8932 | 0.68 | 0.68 | 0.68 | 0.68 | 0.68 |
| L-alanine in 0.30 $\left( {\mathrm{mol}\cdot\mathrm{kg}}^{-1} \right)$ CA | | | | | |
| 0.0000 | 0.50 | 0.51 | 0.52 | 0.52 | 0.53 |
| 0.0858 | 0.67 | 0.67 | 0.67 | 0.67 | 0.67 |
| 0.1864 | 0.67 | 0.67 | 0.67 | 0.67 | 0.67 |
| 0.2857 | 0.67 | 0.67 | 0.67 | 0.67 | 0.67 |
| 0.4147 | 0.67 | 0.67 | 0.67 | 0.67 | 0.67 |
| 0.5290 | 0.67 | 0.67 | 0.67 | 0.67 | 0.68 |
| 0.6150 | 0.67 | 0.67 | 0.67 | 0.67 | 0.68 |
| 0.7023 | 0.67 | 0.67 | 0.67 | 0.68 | 0.68 |
| 0.7884 | 0.67 | 0.67 | 0.68 | 0.68 | 0.68 |
| 0.8430 | 0.67 | 0.68 | 0.68 | 0.68 | 0.68 |
| L-alanine in 0.40 $\left( {\mathrm{mol}\cdot\mathrm{kg}}^{-1} \right)$ CA | | | | | |
| 0.0000 | 0.52 | 0.53 | 0.54 | 0.54 | 0.54 |
| 0.1711 | 0.67 | 0.67 | 0.67 | 0.67 | 0.67 |
| 0.2959 | 0.67 | 0.67 | 0.67 | 0.67 | 0.67 |
| 0.3711 | 0.67 | 0.67 | 0.67 | 0.67 | 0.67 |
| 0.4846 | 0.67 | 0.67 | 0.67 | 0.67 | 0.67 |
| 0.5712 | 0.67 | 0.67 | 0.67 | 0.67 | 0.68 |
| 0.7049 | 0.67 | 0.67 | 0.67 | 0.68 | 0.68 |
| 0.9162 | 0.67 | 0.67 | 0.68 | 0.68 | 0.68 |
| L-alanine in 0.50 $\left( {\mathrm{mol}\cdot\mathrm{kg}}^{-1} \right)$ CA | | | | | |
| 0.0000 | 0.53 | 0.54 | 0.55 | 0.56 | 0.56 |
| 0.0825 | 0.66 | 0.66 | 0.67 | 0.67 | 0.67 |
| 0.1808 | 0.66 | 0.67 | 0.67 | 0.67 | 0.67 |
| 0.2848 | 0.67 | 0.67 | 0.67 | 0.67 | 0.67 |
| 0.3801 | 0.67 | 0.67 | 0.67 | 0.67 | 0.67 |
| 0.4973 | 0.67 | 0.67 | 0.67 | 0.67 | 0.67 |
| 0.6030 | 0.67 | 0.67 | 0.67 | 0.67 | 0.67 |
| 0.7018 | 0.67 | 0.67 | 0.67 | 0.67 | 0.67 |
| 0.7889 | 0.67 | 0.67 | 0.67 | 0.67 | 0.68 |
| 0.8216 | 0.67 | 0.67 | 0.67 | 0.68 | 0.68 |
| Glycine in 0.10 $\left( {\mathrm{mol}\cdot\mathrm{kg}}^{-1} \right)$ SA | | | | | |
| 0.0113 | 0.55 | 0.55 | 0.55 | 0.55 | 0.55 |
| 0.0732 | 0.55 | 0.55 | 0.56 | 0.56 | 0.57 |
| 0.1015 | 0.56 | 0.57 | 0.58 | 0.58 | 0.57 |
| 0.1993 | 0.56 | 0.57 | 0.58 | 0.58 | 0.58 |
| 0.3008 | 0.57 | 0.58 | 0.58 | 0.59 | 0.59 |
| 0.3964 | 0.57 | 0.58 | 0.59 | 0.59 | 0.59 |
| 0.4958 | 0.57 | 0.58 | 0.59 | 0.59 | 0.60 |
| 0.8567 | 0.58 | 0.59 | 0.59 | 0.60 | 0.60 |
| 1.0021 | 0.58 | 0.59 | 0.59 | 0.60 | 0.61 |
| Glycine in 0.30 $\left( {\mathrm{mol}\cdot\mathrm{kg}}^{-1} \right)$ SA | | | | | |
| 0.0711 | 0.48 | 0.51 | 0.57 | 0.57 | 0.58 |
| 0.1041 | 0.52 | 0.52 | 0.57 | 0.57 | 0.58 |
| 0.1963 | 0.54 | 0.55 | 0.58 | 0.58 | 0.59 |
| 0.2951 | 0.56 | 0.56 | 0.59 | 0.59 | 0.59 |
| 03956 | 0.56 | 0.57 | 0.59 | 0.59 | 0.60 |
| 0.4981 | 0.57 | 0.57 | 0.59 | 0.59 | 0.60 |
| 0.8400 | 0.57 | 0.58 | 0.59 | 0.59 | 0.61 |
| 1.0021 | 0.57 | 0.58 | 0.59 | 0.59 | 0.61 |
| Glycine in 0.50 $\left( {\mathrm{mol}\cdot\mathrm{kg}}^{-1} \right)$ SA | | | | | |
| 0.0762 | 0.47 | 0.47 | 0.47 | 0.47 | 0.47 |
| 0.1020 | 0.49 | 0.49 | 0.49 | 0.50 | 0.51 |
| 0.2040 | 0.53 | 0.53 | 0.54 | 0.54 | 0.55 |
| 0.3172 | 0.55 | 0.55 | 0.56 | 0.56 | 0.56 |
| 0.4014 | 0.55 | 0.56 | 0.56 | 0.57 | 0.58 |
| 0.5006 | 0.56 | 0.56 | 0.57 | 0.57 | 0.58 |
| 0.8502 | 0.57 | 0.58 | 0.58 | 0.58 | 0.59 |
| 0.9996 | 0.57 | 0.58 | 0.59 | 0.58 | 0.59 |
